# Characterization of the atmospheric microbiome in a semi-rural area of Central Europe using flow cytometry

**DOI:** 10.1093/ismeco/ycag167

**Published:** 2026-06-16

**Authors:** Ernest Abboud, Pierre Rossi, Benoit Crouzy, Nikolaos Evangeliou, Athanasios Nenes, Kalliopi Violaki

**Affiliations:** Laboratory of Atmospheric Processes and their Impacts, School of Architecture, Civil & Environmental Engineering, École Polytechnique Fédérale de Lausanne (EPFL), CH-1015 Lausanne, Switzerland; Central Environmental Laboratory, School of Architecture, Civil & Environmental Engineering, École Polytechnique Fédérale de Lausanne, CH-1015 Lausanne, Switzerland; Federal Office of Meteorology and Climatology MeteoSwiss, Chemin de l’Aérologie, CH-1530 Payerne, Switzerland; Department for Atmospheric & Climate Research (ATMOS), Stiftelsen NILU (Former Norwegian Institute for Air Research), NO-2027 Kjeller, Norway; Laboratory of Atmospheric Processes and their Impacts, School of Architecture, Civil & Environmental Engineering, École Polytechnique Fédérale de Lausanne (EPFL), CH-1015 Lausanne, Switzerland; Center for the Study of Air Quality and Climate Change (C-STACC), Institute of Chemical Engineering Sciences, Foundation for Research and Technology, GR-26504 Patras, Greece; Laboratory of Atmospheric Processes and their Impacts, School of Architecture, Civil & Environmental Engineering, École Polytechnique Fédérale de Lausanne (EPFL), CH-1015 Lausanne, Switzerland

**Keywords:** atmospheric microbiome, bioaerosols, flow cytometry, bacteria, fungi, pollen, Payerne

## Abstract

Characterizing bioaerosols is important for understanding their potential impacts on the environment and public health. In this study, we developed a novel flow cytometry-based approach to determine the low nucleic acid (LNA), high nucleic acid (HNA), dead, and intact bioaerosol populations in samples collected with a wet cyclone at Payerne, Switzerland, during spring and summer 2024. We found that the average bioaerosol number concentration reached (2.47 ± 3.35)×10^4^ m^−3^. The HNA and intact populations were the most abundant populations, representing the largest fraction of total bioaerosols within 65% and 97% of the samples, respectively. Our results show that the LNA can be composed of dead bioaerosols, which correlated strongly with atmospheric particulate mass. Quantitative Polymerase Chain Reaction (qPCR) and metagenomic analysis reveal significant correlations and associations (Spearman, PERMANOVA, and Mantel) between the different kingdoms analyzed, reflecting complex ecological interactions in the atmosphere among the communities. Despite this complexity, LNA was mainly associated with the archaea *Nitrososphaerota* and bacteria *Actinomycetota*, whereas HNA was enriched by fungal classes such as *Pichiomycetes* and *Ustilaginomycetes*. Pollen abundance was positively correlated with temperature and negatively correlated with relative humidity and pollution (NO_x_ and NO_2_), as these conditions promote the formation of sub-pollen particles (pollen fragments) through osmotic (bursting) and oxidative stress. Factor analysis indicates a seasonal dynamics transition from plant-associated bioaerosols in the spring season, to other bioaerosol types to be co-emitted during summer. Overall, the integration of flow cytometry with molecular analysis provides a framework to characterize and quantify bioaerosols and provides new insights into the ecological structure, variability, and sources of the atmospheric microbiome.

## Introduction

Bioaerosols, or airborne biological particles, range in size from a few nanometers (e.g. viruses) to a dozen micrometers (e.g. pollen), and they comprise of bacteria, fungal spores, archaea, protists, plant, cell fragments, and extracellular material. Compared to other ecosystems, bioaerosols are found at much lower abundances (atmosphere: 10^2^–10^6^ m^−3^, soil: 3 × 10^14^ m^−3^, and oceans: 10^9^ m^−3^) [1–5]. Bioaerosols form ice at very warm temperatures and can have an important impact on cloud formation, precipitation, the hydrological cycle, and climate (e.g. [6–9]). They are major contributors to the biogeochemical cycle of phosphorus [10], contributing upon deposition, significantly to the external fertilization of the oligotrophic marine environments (e.g. Eastern Mediterranean). Bioaerosols have been found attached to dust particles [11–13] and undergo long-range transport across continents and oceans, facilitating their dispersal and beyond their environmental impact. Such events influence both human health by promoting allergic and oxidative stress responses [11, 12] and microbial ecology by increasing the local richness and diversity of airborne communities during dust periods compared to non-dusty periods [14–16].

Characterizing and quantifying bioaerosols relied on culturing them and counting the number of colonies formed per unit [1]. However, this approach has limitations as it only covers an estimated 5% of bacteria and 17% of fungi that can be cultured in the lab after collection from the atmosphere [1, 17–20]. This method cannot quantify viable but non-culturable and dead microorganisms or viruses. Techniques, such as epifluorescence microscopy, allow for the counting and some characterization of bioaerosols but are labor-intensive, subject to counting errors and biases, and therefore not suitable for a large number of samples [2, 21]. Negron *et al*. and Liang *et al.* [2, 22] provided an alternative approach, based on flow cytometry (FCM), to characterize and quantify bioaerosols in Atlanta, Georgia, USA and in Beijing, China, respectively. Negron *et al*. [2] identified two populations after the usage of DNA stains: low nucleic acid (LNA) and high nucleic acid (HNA) particles, hypothesized to represent less metabolically active bacterial cells and fungal spores, respectively.

While these studies provided important insights, their conclusions were based on a single nucleic acid stain, limiting the ability to differentiate physiological states and population functions. However, flow cytometry has the potential to go beyond single staining and cellular quantification. The combination of multiple dyes targeting different cellular properties; e.g. Propidium iodide (PI) enters only the cells with damaged membranes and SytoX the cells with non-damaged membranes, or the reduced carboxyfluorescein diacetate (also known as CFDA) being oxidized via enzymatic activity to evaluate the metabolic state of the cell [23]; offer a multidimensional understanding of bioaerosol viability and functionality in the atmosphere under changing conditions (oxidation, acidification, etc). Understanding these aspects of the bioaerosol populations is essential for assessing their influence on atmospheric processes. For example, HNA and LNA could potentially differ in their ability to participate in key atmospheric processes such as nucleate ice and thus modulate cloud formation.

This study aims to characterize bioaerosols by their identification and quantification using FCM to understand the population variability during spring and summer in a typical semi-rural area in Central Europe (Payerne, Switzerland). A combination of fluorescent dyes was used to identify the LNA, HNA, and plant populations, as well as to distinguish between intact and non-viable (dead) cells. Taxonomic identification of selected samples was achieved by applying the Oxford Nanopore Technologies (ONT) sequencing, while qPCR was used to estimate the bioaerosol abundance. In addition, meteorological parameters and atmospheric pollutants were included in the analysis to provide a deeper characterization of these populations.

## Materials and methods

### Sample collection

**Figure 1:**
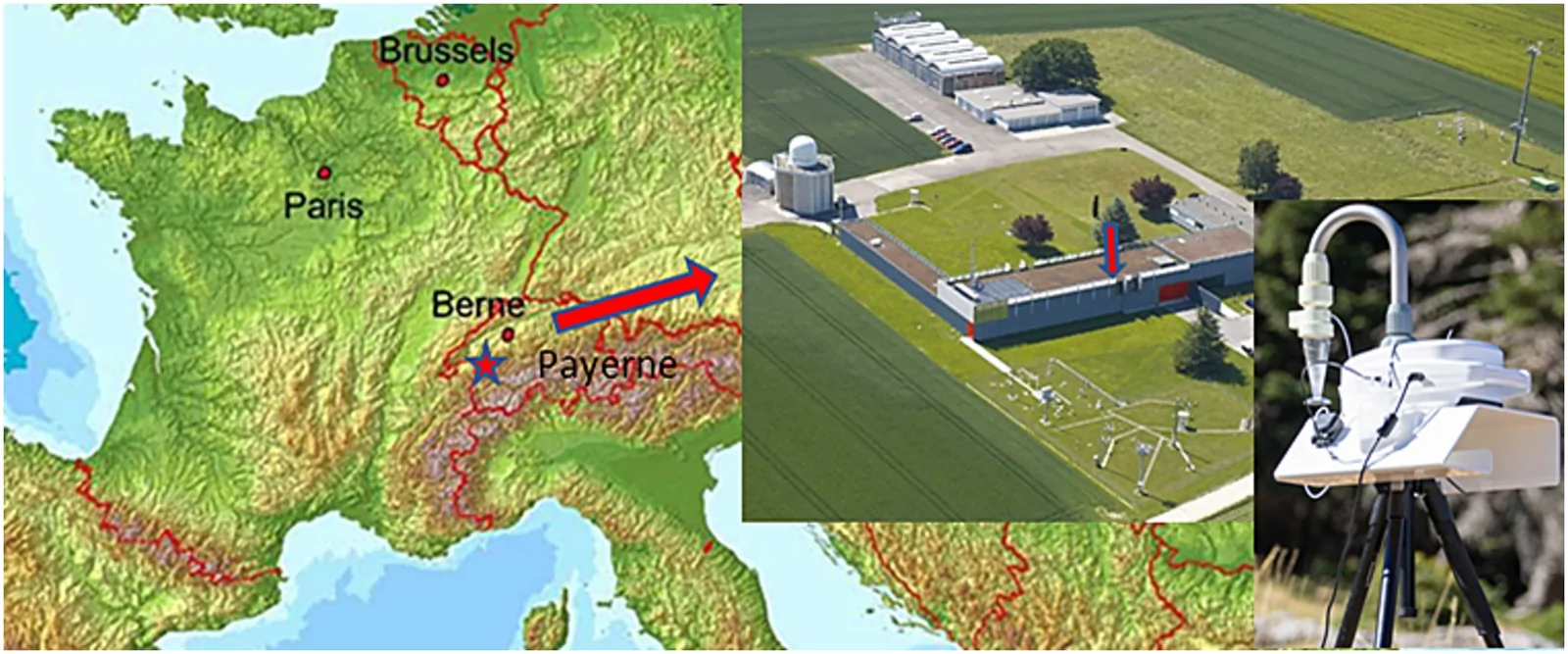
Map of the sampling site, located in central Europe (Switzerland). The samples (*n* = 37) were collected on the roof of the MeteoSwiss Payerne station (46.81N, 6.94E, 490 m a.s.l.), a rural site surrounded by agricultural fields, from 25 March to 26 August 2024, using a Coriolis µ sampler.

A total of 37 samples were collected on the roof of the MeteoSwiss Payerne station (46.81N, 6.94E, 490 m a.s.l.), a rural site surrounded by agricultural fields, from 25 March to 26 August 2024 (Fig. 1). A portable wet cyclone (Coriolis μ, Bertin Technologies, www.bertin-technologies.com) was operated at a nominal flow rate of 300 L min^−1^ for 3 h. Phosphate-buffered saline solution (PBS 1×, pH = 7.4, Gibco) was used as the collection medium, with evaporation compensated by replenishing the liquid to maintain a volume of ~10 mL in the cone. The cone was changed every hour to increase the collection efficiency, minimize re-aerosolization, and flow-induced stress on cells, which can compromise cell viability [24] (SI 3.1, Fig. S10, Table S11).

After sampling, the cones were pooled and 5 mL was transferred into 15 mL Falcon tubes for FCM, fixed with 0.74% of formalin (37% wt formaldehyde, ThermoFisher), and stored at 4°C (SI 3.2, Fig. S11, Table S12). This concentration was selected after a sensitivity test performed using only PBS and formalin to evaluate background particle formation. Interestingly, 0.74% yielded a lower background signal during FCM analysis compared to the 0.37% used [2] and to the 2% used [22] (919 ± 84.67, 1917 ± 491.83, and 1257 ± 176.47 particles, respectively, *n* = 5). The remaining liquid was frozen at −80°C for DNA extraction. Field blanks were included by processing the PBS from the sampling refill bottle alongside the samples (*n* = 30). To minimize the risk of contamination between sampling, the air intake, canes, cones, and refill bottle were cleaned three times with ultrapure water (Millipore, USA), followed by a wash with 70% ethanol, and three additional rinses with ultrapure water. The air intake and cane were dried and wrapped in aluminum foil, while the cones and refill bottles were filled with PBS and sterilized by autoclaving using a predefined program at 126°C and 2.5 bar for 20 min.

### Flow cytometry

#### Instrumentation and workflow

An Attune™ CytPix™ Flow Cytometer (ThermoFisher), equipped with an integrated light microscope, was used for bioaerosol quantification and imaging, acquiring a maximum of 15 000 pictures per analysis. Five of the 14 lasers were calibrated according to the manufacturer: FSC-A (forward scattering) for particle size, the SSC-A (side scattering at 90°) to assess internal complexity [25], BL1-A laser (530 ± 30 nm) for Syto13 detection, YL2-A laser (620 ± 15 nm) for PI detection, and RL1-A (670 ± 14 nm) for pollen detection due to the natural fluorescence of the chloroplast in this spectrum [26] and the beads. Particles below the detection limit of 0.30 μm were excluded, while those above 33.63 μm were included but capped at 33.63 μm, corresponding to the upper detection limit.

Particle size was determined using calibration beads of known size of 1.0, 2.0, 4.0, 6.0, 10.0, and 15.0 μm (Flow Cytometry Size Calibration Kit (nonfluorescent microspheres, https://www.thermofisher.com/order/catalog/product/F13838) (Fig. S2C). The equation was determined after performing a linear regression:


\begin{equation*} \mathrm{d}\ = \frac{FSC\!-\!A\ _{intensity}+31\ 000}{32\ 100} \end{equation*}

Where d is the particle size (μm) and *FSC-A _intensity_* represents the intensity of the forward scattering area. Samples were incubated with 2.5 μM of Syto13, 3.0 μM of PI, and 10 μL of a known concentration of autofluorescent beads for quantification (CountBright™ Absolute Counting Beads, ThermoFisher) in the dark for 15 min before analysis [27] and analyzed at the determined optimal flow rate of 200 μL min^−1^. Before each run, the instrument was cleaned with 10% bleach and flushed with 0.1 μm-filtered water until background counts were below 0.01 particles μL^−1^ at the highest flow rate (1000 μL min^−1^). The flow cytometer was cleaned between every run with 1 mL of filtered distilled water. All the FCS files acquired were analyzed on FlowJo (last accessed: 23 November 2024). FlowSOM, PhenoGraph, and XShift plugins were downloaded from Plugin Exchange | FlowJo, LLC on 29 August 2024.

A standard of olive pollen (*Olea europaea L.*) was purchased from Bonapol (https://www.bonapol.com/) to quantify the generation of sub-pollen particles in contact with PBS. An inactivated mixture of 8 bacteria and 2 fungi (ZymoBIOMICS™ Microbial Community Standard, Zymo, USA) was used as a standard for those microorganisms.

The FCM does not have a perfect counting efficiency; an internal standard (autofluorescent beads) was used to correct all bioaerosol populations by multiplying the concentration of each population by the expected-to-observed beads concentration [21]. The flow rate value was set to 270 L min^−1^ as found in [24].


(1) \begin{eqnarray*} R=\frac{B}{C} \end{eqnarray*}

Where *R* is the ratio of theoretical to counted beads, *B* represents the number of fluorescent beads added according to the manufacturer, and *C* is the number of beads detected by the FCM.


(2) \begin{eqnarray*} Nb=\frac{\left( PF- C- Pb\right)\times R}{Va} \end{eqnarray*}

Where *Nb* is the number of bioaerosols per μl, *PF* is the total number of particles counted by the FCM, *C* is the number of beads detected, *Pb* is the number of background particles detected in the blank, *R* is the bead ratio defined in Eq. (1), and *Va* is the sample volume analyzed by the instrument (in μL).


(3) \begin{eqnarray*} Nb\ adjusted=\frac{Nb\times 10^{3}\times Vf}{Q\times 10^{-3}\times t} \end{eqnarray*}

Where *Nb\ adjusted* is the number of bioaerosols per cubic meter of air (m^−3^), *Nb* is the number of bioaerosols per μl found in Eq. (2), *Vf* is the final liquid volume of the sample (mL), *Q* is the flow rate of the sampler (L min^−1^), and *t* is the sampling duration (min).

#### Validation of clustering algorithm for bioaerosol populations

To determine the most suitable clustering algorithm, the three best-performing ones, FlowSOM, PhenoGraph, and XShift, were tested on every acquired FCM file [24]. For PhenoGraph and XShift, the default proposed k-value was kept, as it is difficult and time-consuming to determine the optimal value for each FCM file due to the events variability in each sample. FlowSOM requires specifying only the number of clusters returned, which was set to 40 [24]. PhenoGraph and XShift failed to generate output clusters for 3 and 1 samples, despite minimal input of 84 710 and 212 957 events measured by FCM, respectively, whereas FlowSOM performed well on all datasets, supporting its suitability for atmospheric FCM analysis (Table S1).

The distribution of bioaerosols on cytoplots was assessed using standards for bacteria and fungi (Zymo), and for pollen (*O. europaea L.*) without and with the addition of Syto13 and PI (Fig. S1A–E). Autofluorescence of the Zymo standard ranged between 10^1^ and 10^2^ arbitrary units (AU) across BL1-A and YL2-A fluorescence lasers (Syto13 and PI, respectively), whereas the beads displayed higher autofluorescence, facilitating their distinction (Fig. S1A and B). After running FlowSOM, the 99^th^ percentile was calculated for Syto13 and PI to establish the baseline threshold for nucleic acid staining. After the addition of dyes, the output clusters from FlowSOM above the Syto13 threshold were merged to form the HNA population (Syto13^+^), and those below formed the LNA population (Syto13^−^). PI vs Syto13 cytoplots showed the HNA population with occasional double-staining (Syto13^+^/PI^+^), likely due to partial permeabilization of the standard, while no events were detected in the dead quadrant (Syto13^−^/PI^+^) (Fig. S1C and D). Pollen autofluorescence was above 10^3^ AU across all lasers used due to the presence of fluorochromes, such as chlorophyll (Fig. S1E and F). Several pollen fragments were present between 10^1^ and 10^2^ AU, which are referred to as sub-pollen particles (SPPs) (Fig. S1E and F). The SPPs quantification in 0.01 mg of olive pollen represented 22% of the total particles counted. Calculations of olive pollen number and SPPs, considering a mean diameter of 20 ± 3 μm [28] and a density of 1.0 g cm^−3^ [29], showed that each pollen generated ~5 SPPs. Being 100 to 1000 times less abundant than bacteria and fungi in the lower atmosphere, 117 times less in this study, SPPs minimize their influence on the total bioaerosols quantification [1, 29]. Following the addition of dyes, both Syto13 and PI entered pollen, and the Syto13^+^/PI^+^ from the pollen population did not interfere with bacteria and fungi from Zymo, forming two distinct populations (Fig. S1G–L).

The workflow was then applied to atmospheric samples, and the fluctuation of the geometrical mean of each population was analyzed on 10 samples collected consecutively (Fig. S2A). Syto13 intensity consistently exceeded PI intensity in all populations, except the sample collected on 20^th^ July for the LNA and dead populations. While fluctuations within the LNA and dead populations were minimal (<550 AU), the ones in the HNA and pollen populations showed an 8- and 4-fold difference, respectively. The Shapiro–Wilk test indicated a normal distribution of the geometrical mean across the populations, except for SSC-A in HNA and PI in pollen (*P* < .05) (Fig. S2B). Overall, this FCM workflow enabled the reliable detection and quantification of the different populations in both the representative standards and atmospheric samples.

### DNA extraction

DNA was extracted from 31 samples and their 31 corresponding blanks (*n* = 62, all samples collected after 22/04/2024). Swinnex filter holders were fitted with 0.22 μm PES membranes (Millipore), wrapped in a paper towel, and autoclaved (30 min, 121°C). Samples were thawed at room temperature and filtered using a sterile syringe on the membrane. The latter was placed into a PowerBeads extraction tube with 1.2 mL of sterile CD1 solution (Qiagen, USA), heated at 55°C for 5 min, and then placed in the bead-beating device (Precellys 24, Bertin, France). Samples were homogenized twice at 5500 rpm for 30 s with a 30 s pause and centrifuged at 10 000 g for 1 min (Eppendorf 5424 centrifuge). The supernatant (600 μL) was transferred into a rotor adapter, run in a Qiacube Connect robot in ISR mode, and eluted into 60 μL of EB buffer (Qiagen, USA). DNA quantification was performed using a fluorescent technique (Qubit dsDNA HS Assay kit, Invitrogen, USA).

### Quantitative PCR analysis (qPCRs)

The prokaryotic 16S rRNA gene and the eukaryotic ITS and 18S rRNA genes were targeted using qPCR (Table S2). Reactions were performed in a MIC qPCR Cycler (BioMolecular Systems, Australia) in 10 μL volumes as follows: 2.5 μL of DNA; 0.2 μM of each primer; and 1× SensiFast SYBR® No-ROX Kit (Meridian Bioscience, England), topped up with water.

Samples were cycled 40 times at 95°C for 5 s, followed by extension at kingdom-specific temperature (Table S2) for 15 s, and acquisition at 72°C for 15 s. The final melting step was performed from 72°C to 95°C at a rate of 0.1°C/s. Analysis of the results was performed using the integrated analytical software (Workbench v2, BioMolecular Systems, Australia). On average, efficiency (0.74–1.01) and r^2^ values (>0.97) were determined from seven points of serial dilutions (10^7^–10^1^ copies) of the target gene.

### Oxford Nanopore Technologies sequencing, phylogenetic analysis, and statistical analysis

The full 16S or ITS and 18S rRNA genes were amplified for each kingdom (Table S2) in 20 representative samples of the sampling period. For all PCRs, a 25 μL reaction was performed as follows: 5 μL of DNA template, 0.8 μM of each primer, 12.5 μL of LongAmp™ Taq 2× Master Mix (New England Biolabs, UK), and filled with water. Samples were cycled 35 times at 95°C for 30 s, followed by annealing at kingdom-specific temperature (Table S2) for 15 s and extension at 65°C for 75 s.

PCR products were purified, and 100–200 fmol from each kingdom were pooled by sample into a 96-well plate for ONT barcoding following the manufacturer's guidelines. After barcoding, samples were purified before the End-prep reaction (Ultra II End-prep Enzyme Mix) and adapter ligation (NEBNext Quick T4 DNA Ligase, both NEB, UK), following the manufacturer's instructions. Sequencing was performed on a MinION sequencer using R9.4.1 FLO-MIN 106 flow cells for up to 48 h.

The raw pod5 data were highly accurate basecalled using Dorado v0.9.6, converted into BAM files, then FASTQ, demultiplexed, and trimmed using samtools v1.21 [30]. Reads were filtered applying a Phred score of 9 using fastplong v0.2.2 [31], demultiplexed by Kingdom, and aligned to their respective database with cutadapt v4.4 and kraken2 v2.1.5, respectively [32, 33].

All the statistical analyses were performed using R (ver. 4.4.0) [34]. *P*-values below .10 were considered statistically significant. Factor analysis for source apportionment was performed using XLSTAT 2024.1 (Addinsoft, Boston, USA).

### Meteorological and air quality data

Air quality data (PM_2.5_, PM_10_, SO_2_, NO_2_, O_3_, CO, EC, NO_x_) were obtained from the National Air Pollution Monitoring Network (NABEL: https://www.bafu.admin.ch/bafu/fr/home/themes/air/pollution-de-l-air/donnees/requete-de-donnees-nabel.html; latest access: 6 February 2025). Meteorological data (Wind speed, sunshine (hours of sun), relative humidity (RH), temperature, radiation, precipitation) and pollen concentrations (m^−3^) were obtained from MeteoSwiss (Payerne, Open Data - MeteoSwiss; latest access: 7 February 2025). All data were adapted to the sampling period and reported as an average minus/plus standard deviation. MeteoSwiss classifies pollen manually into 48 taxa counted by microscopy (more information: Pollen monitoring network - manual method - MeteoSwiss).

FLEXPART 11 [35] (https://atmo-access.nilu.no/PAYERN_HRDU.py) was applied in backward (retroplume) mode to determine the potential source regions and transport pathways of air masses carrying fine and coarse dust arriving at the sampling site up to 50 m. FLEXPART 11 is a Lagrangian particle dispersion model driven by assimilated meteorological fields (ERA5, [36]) to simulate the atmospheric transport, turbulence, gravitational settling, wet/dry deposition, and diffusion of tracers such as dust. The model tracks a large number of particles backward in time and calculates footprint emission sensitivities (often called source-receptor relationships), which express the probability of any hypothetical emission to arrive at the receptor.

## Results

### Characterization and quantification of bioaerosols using flow cytometry

**Figure 2:**
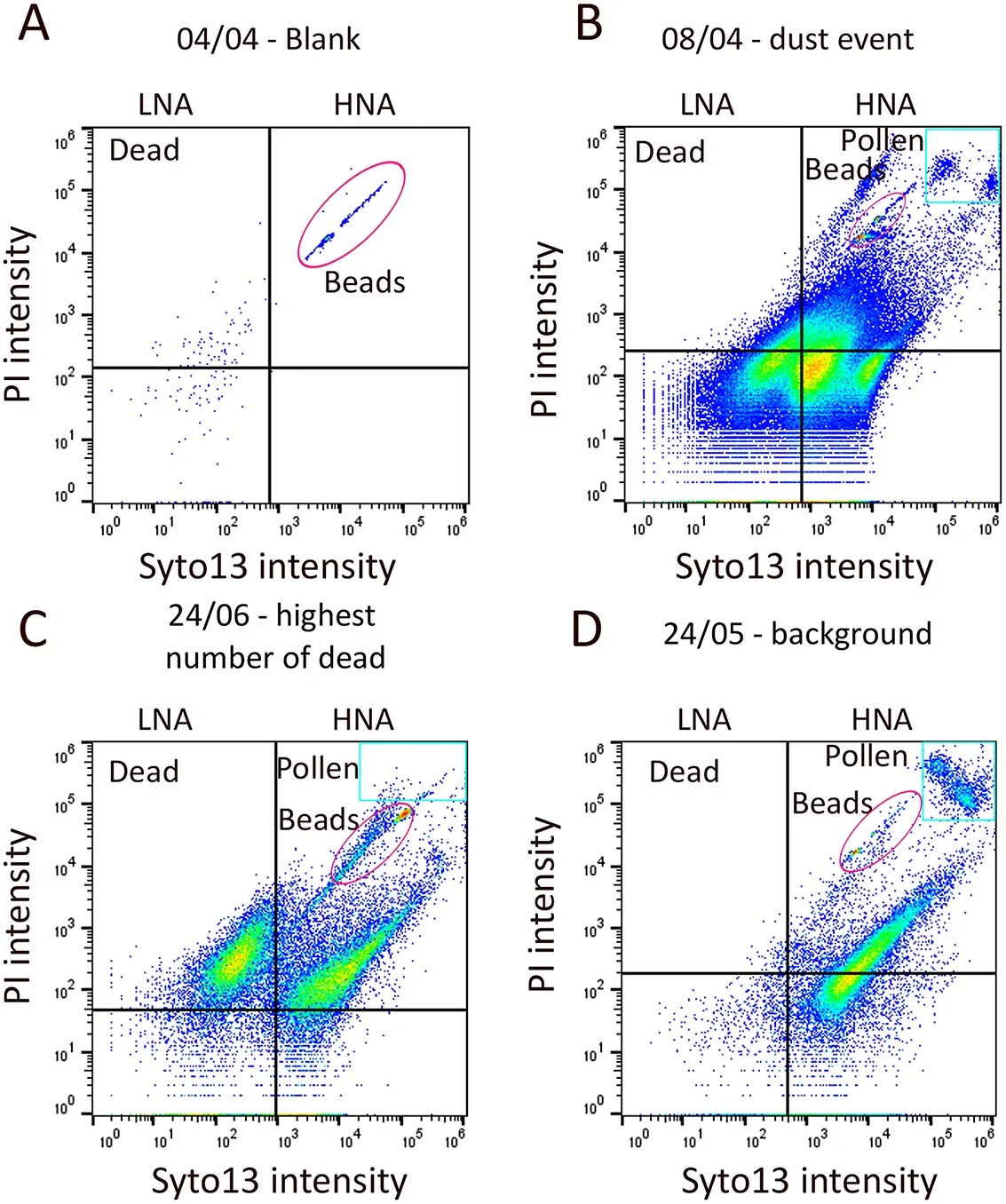
Cytoplots representing PI intensity (YL2-A) vs Syto13 intensity (BL1-A) in AU (arbitrary units) of **(A)** a blank with a few events, represented by the dots and the beads (internal standard) (4 April 2024; 04/04), **(B)** a dust event (8 April 2024; 08/04), **(C)** the day with the highest number of dead bioaerosols showing two distinct populations: one in the dead quadrants and another in the intact/HNA quadrants (24 June 2024; 24/06), **(D)** and a background (or average), typical for the sampling period (24 May 2024; 24/05). Quadrants are the threshold: below the Syto13 threshold is the LNA population and above the HNA one. On the top left quadrant (Syto13 negative and PI positive) is the dead population. The intact population is represented by the rest of the quadrants (without the beads; internal standard). The circle represents the beads identified by the workflow developed in section 2.2.2 and the rectangle represents the pollen population.

**Figure 3:**
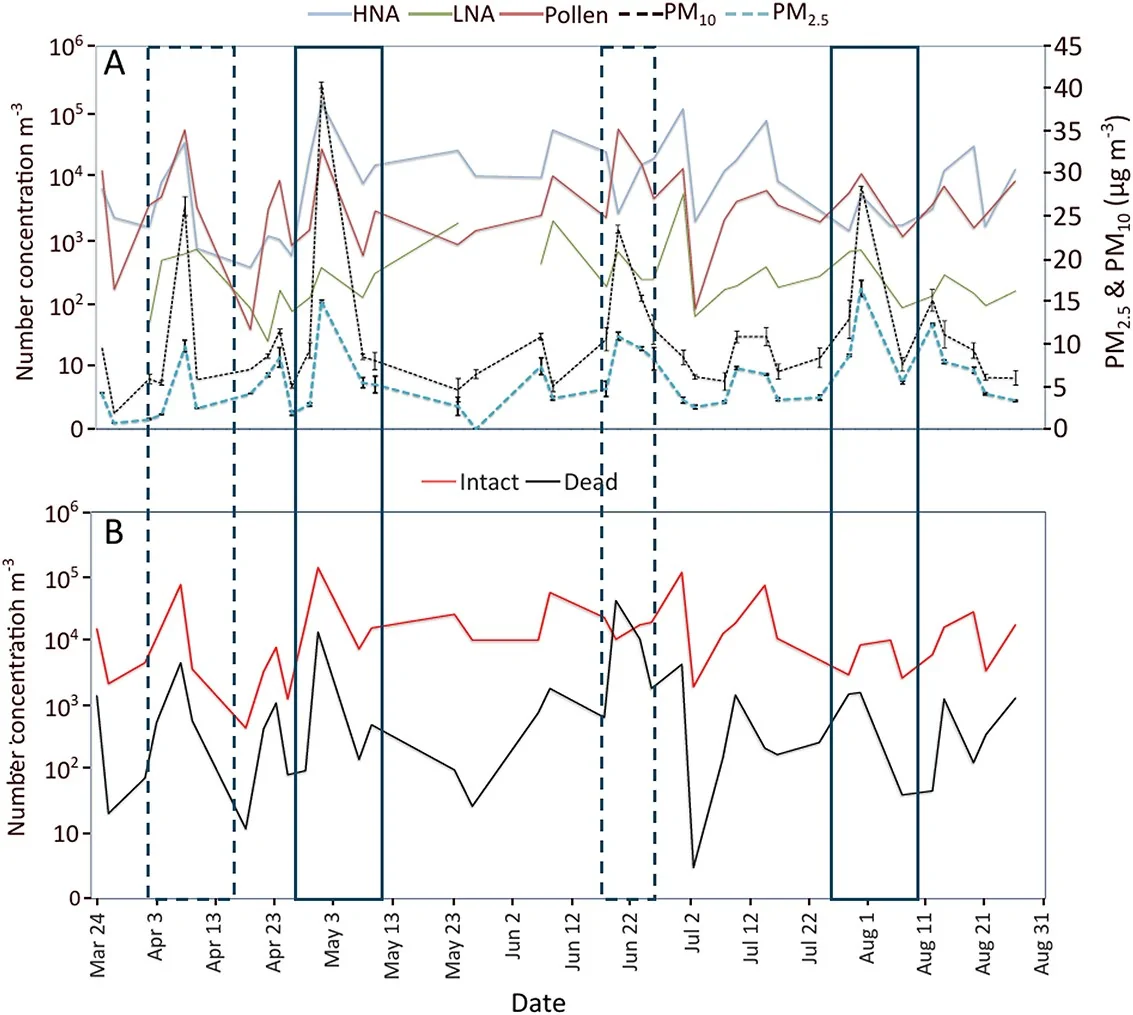
Number concentrations (m^−3^) of the different populations identified with FCM during the sampling period (from 25 March 2024 to 26 August 2024) in Payerne, Switzerland **(A)** LNA, HNA, and pollen with PM_10_ (μg m^−3^) and PM_2.5_ (μg m^−3^) in dashed line (error bars show the standard deviation) and **(B)** intact and dead populations. The dashed frames indicate the Saharan dust events (*n* = 2) recorded during the sampling period (Fig. S3) and the solid frames indicate the mixed air masses transported from the east or west part of Europe (*n* = 2, Fig. S3).

**Figure 4:**
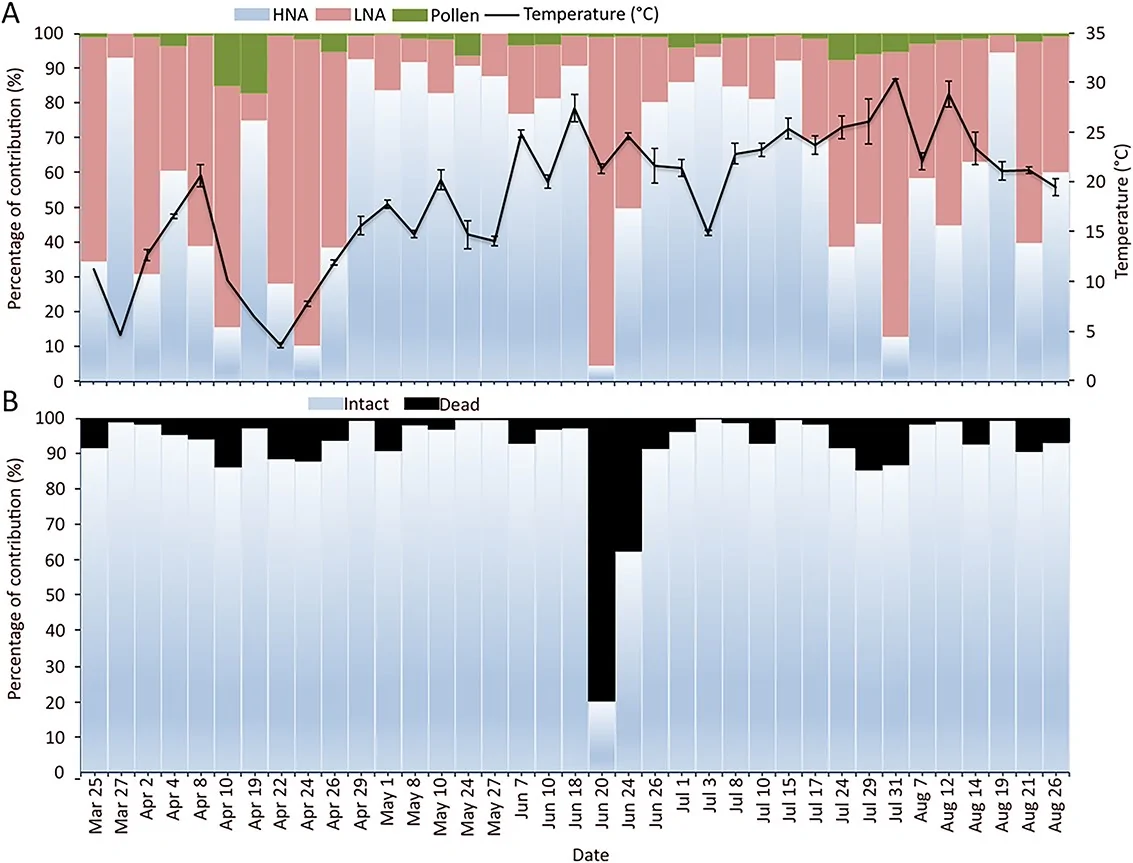
Percentage contribution of the **(A)** LNA, HNA, and pollen populations, and **(B)** intact and dead populations for each sample to total microorganisms measured with FCM. Error bars represent the standard deviations in temperature measurements.

Application of the validated clustering workflow to the analyzed samples yielded an average of (2.47 ± 3.35) × 10^4^ biological particles m^−3^, ranging from 4.60 × 10^2^ to 1.53 × 10^5^ m^−3^ (Fig. 2 and Fig. 3). Excluding dust event samples (*n* = 2) reduced the numbers by 9.8 ± 2.5% (Fig. S3). The HNA population averaged (1.72 ± 2.79)×10^4^ biological particles m^−3^, 145% higher than the LNA population (7.02 ± 11.4) × 10^3^ m^−3^, and was dominant in 65% of samples (Fig. 3 and Fig. 4).

The size distribution of the HNA population, characterized by the presence of large particles, indicated a dominance of the particles of 1.5–4 μm (average size of 4.0 ± 3.4 μm, reaching a peak at 1.8 μm) while the LNA population by particles of 1.5–3 μm (average size of 2.7 ± 1.8 μm, reaching also a peak at 1.8 μm) (Fig. S4A and B, Table S3). The size distribution of the pollen collected in our study ranged from 8.0 (e.g. small pollen such as those from some species of the Poaceae family) to 33.6 μm (e.g. large pollen such as that of the Fabales family) (average size of 16.4 ± 9.3 μm), within the pollen size range [37, 38] (Fig. S4C, Table S4). The size distribution of the intact particles is dominated by particles of 1.5–3 μm size (average size of 3.9 ± 3.8 μm, reaching a peak at 1.8 μm), larger than the ones in the dead population by particles of 1.8–2.5 (average size of 2.5 ± 1.4 μm, reaching a peak at 1.8 μm) (Fig. S4D and E, Table S3). Most samples showed a unimodal distribution, whereas seven samples displayed a bimodal distribution in the HNA and five in the intact populations, suggesting the presence of two distinct types of bioaerosols (Fig. S4).

In addition to having the same size distribution and average size, a strong correlation was observed between the LNA and the dead populations (r_s_ = 0.89, *P* < .001 versus r_s_ = 0.41 with the intact populations, *P* > .10) and between the HNA and intact populations (r_s_ = 0.96, *P* < .001 versus r_s_ = 0.28 with the dead population, *P* > .10). LNA correlated positively with PM_2.5_ and PM_10_ (r_s_ = 0.62 and 0.59, respectively, *P* < .001); dead cells showed similar correlations (r_s_ = 0.52 and 0.50, respectively, *P* < .001). HNA and intact populations did not correlate with either PM_2.5_ or PM_10_ (r_s_ = −0.07, 0.03, 0, and 0.10, respectively, *P* > .10), or any other atmospheric variable measured. However, pollen was found to be negatively correlated with RH (r_s_ = −0.37, *P* < .001) and with NO_x_ and NO_2_ (r_s_ = −0.47 and − 0.41, respectively, *P* < .05) but positively with temperature and radiation (r_s_ = 0.37 and 0.32, respectively, *P* < .001), which could be influenced by the sampling time (Fig. S5). Interestingly, no population showed a significant correlation with precipitation.

### Biomolecular characterization of bioaerosols using qPCR and ONT sequencing

**Figure 5:**
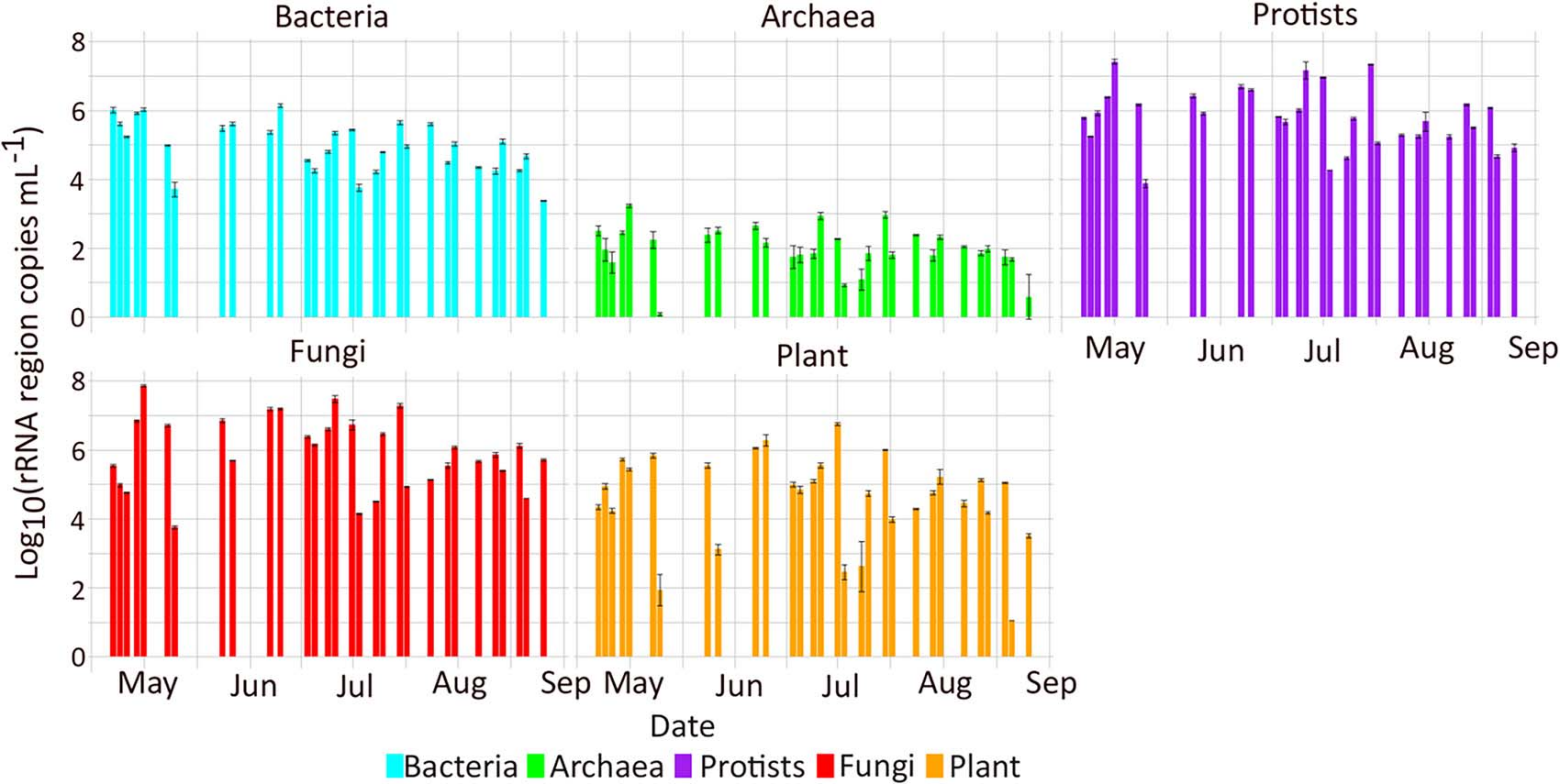
Quantification of the rRNA genes region by qPCR in the bacterial, archaeal, protist, fungal and plant kingdoms collected during the sampling period (from 25 March 2024 to 26 August 2024). Error bars represent the standard deviation.

The presence of multiple kingdoms was confirmed by qPCR in the samples. Among the prokaryotes, bacteria [(2.64 ± 3.58) × 10^5^ copies mL^−1^ of sample] were ~1000-fold more abundant than archaea with a count of (2.38 ± 3.62) × 10^2^ copies mL^−1^ of sample (Fig. 5 and Table S5). Among the eukaryotes, protists [(3.24 ± 6.49) × 10^6^ copies mL^−1^ of sample] and fungi [(6.45 ± 14.0) × 10^6^ copies mL^−1^ of sample] showed similar levels of abundance, while plant particles [(4.34 ± 10.8) × 10^5^ copies mL^−1^ of sample] were ~10 times less. The field blank showed a reading that was at least two orders of magnitude lower than that of its corresponding field samples with no detectable contamination in several archaeal and plant samples (Fig. S6 and Table S5).

A significant positive correlation between the pollen population identified by FCM and plant sequences (r_s_ = 0.47, *P* < .001) was observed, further supporting the validity of the clustering method. Strong correlations among kingdoms quantified by qPCR were identified (0.57 < r_s_ < 0.80, *P* < .05), supported by further analysis with the Mantel test, identifying significant associations between the kingdoms (Table S6). Bacteria associated with protists, plants, and fungi (r__M__ = 0.90, 0.24, and 0.23, respectively, *P* < .10), while fungi associated well with archaea and protists (r__M__ = 0.43 and 0.19, respectively, *P* < .10). The HNA population strongly correlated with plants, fungi, and bacteria qPCR copy numbers (r_s_ = 0.47, 0.70, and 0.57, respectively, *P* < .05) and associated with the bacterial and archaeal communities based on PERMANOVA analysis (r__PERMANOVA__ = 0.15 and 0.26, respectively, *P* < .05) (Fig. S8 and Table S7). Bacterial and archaeal communities were significantly associated with the LNA population (r__PERMANOVA__ = 0.16 and 0.27, respectively, *P* < .05). The fungal community also showed associations with atmospheric data such as RH, NO_x_, NO_2_, SO_2_, CO, and PM_10_ (r__PERMANOVA_ _= 0.13, 0.23, 0.22, 0.11, 0.11, and 0.13, respectively, *P* < .10).

**Figure 6:**
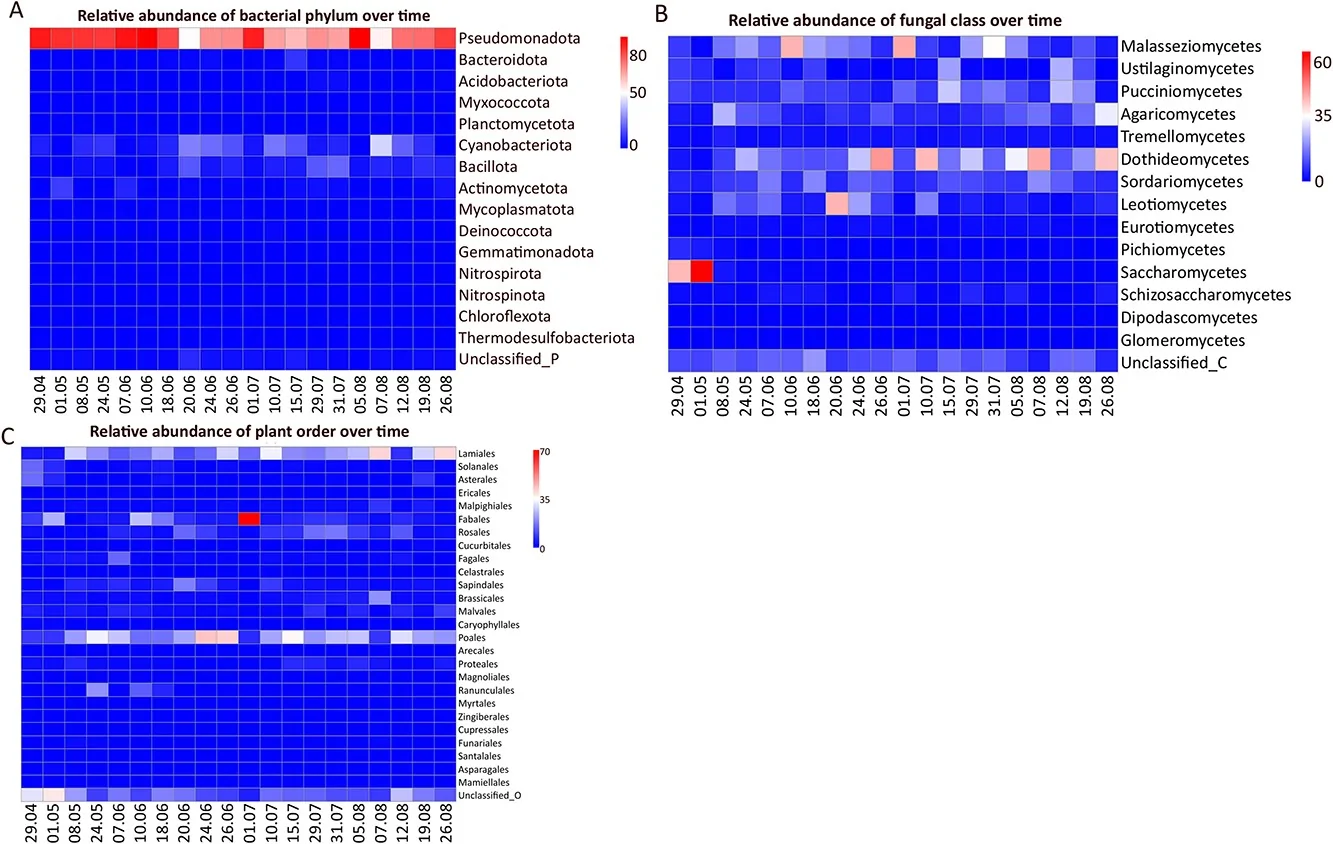
Taxonomic identification of a selection of 20 samples after Oxford Nanopore Technologies (ONT) sequencing at **(A)** the phylum level for bacteria, **(B)** the class level for fungi, and **(C)** the order level for plants. Unclassified_P represents sequences unassigned at the phylum level, Unclassified_C corresponds to sequences unassigned at the class level, and Unclassified_O includes sequences unassigned at the order level.

To determine the bioaerosol composition, 20 representative samples were selected for ONT sequencing. The analysis yielded over 22 million reads, of which 4.7% did not pass quality control. Of the high-quality reads, 93.4 ± 10.5% were classified at least at the phylum level, while 6.6 ± 10.5% remained unclassified (Table S8). All kingdoms had a high rate of classification (above 95%) except for the archaea, with only 18.9 ± 13.1% of classified sequences (Table S8). ONT sequencing revealed that at the phylum level, the archaeal communities were dominated by *Thermoplasmatota* in 50% of samples and accounted for 10.1% ± 8.5% of the total archaeal sequences, followed by *Nitrososphaerota* (6.9% ± 11.0%) and *Methanobacteriota* (0.2 ± 0.4%) (Fig. S7A). Three dominant phyla were detected in bacterial communities: *Pseudomonadota* (76.1 ± 14.1%), *Bacillota* (6.2 ± 5.6%), and *Actinomycetota* (2.0 ± 3.0%) (Fig. 6A) [1]. Also, photosynthetic bacterial phyla were detected: *Chloroflexota* and *Cyanobacteriota*, accounting for 11% of the total bacterial reads. The fungal phyla were dominated by *Ascomycota* and *Basidiomycota* (95.8 ± 2.8%). At the class level (Fig. 6B), both pathogenic and non-pathogenic fungi were identified, such as *Dothideomycetes* (20.0 ± 13.9%), *Malasseziomycetes* (15.2 ± 12.2%), *Pucciniomycetes* (10.1 ± 6.5%), and *Agaricomycetes* (9.3 ± 7.0%) [39–41]. Protist phyla were dominated by *Bacillariophyta* (43.5 ± 13.0%), *Oomycota* (32.0 ± 13.8%, several species are plant pathogens), and *Apicomplexa* (12.8 ± 14.9%) (Fig. S7B). Finally, the plant kingdom was dominated by *Streptophyta* and *Magnoliopsida* at the phylum and class level, respectively, accounting more than 97% of the reads. A higher diversity emerged at the order level with the presence of 26 orders, of which 13 individually contributed to ˂1% of the total reads (Fig. 6C). Spearman correlation analysis indicated that the LNA population was strongly positively correlated with the archaeal phylum *Nitrososphaerota*, the bacterial phylum *Actinomycetota*, and the plant order Fabales (r_s_ = 0.48, 0.40, and 0.57, respectively *P* < .10), and negatively correlated with the fungal classes *Eurotiomycetes* and *Agaricomycetes* and the plant order Lamiales (r_s_ = −0.47, −0.45, and − 0.47, respectively, *P* < .05). The HNA population showed positive correlations with the fungal classes *Pichiomycetes*, and *Ustilaginomycetes*, the protist phylum *Apicomplexa*, and the unidentified protists (r_s_ = 0.40, 0.49, 0.40, and 0.43, respectively, *P* < .10) and negative correlations with the fungal class *Agaricomycetes, Eurotiomycetes*, the bacterial phyla *Bacillota*, and the plant orders Brassicales, Celastrales, and Lamiales (r_s_ = −0.60,  −0.40, −0.45, −0.62, −0.57, and − 0.40, *P* < .10) (Fig. S8). PERMANOVA analysis indicated a significant difference in bioaerosol composition between spring (April and May, *n* = 4) and summer (June–August, *n* = 16), with the strongest seasonal differentiation from bacteria (r__PERMANOVA__ = 0.26, *P* < .05) (Table S7).

## Discussion

Traditionally, FCM data were analyzed using manual gating on a 2D scatter plot, a method limited by human biases and poor reproducibility, particularly for high-dimensional datasets in which the non-represented dimensions are ignored [42, 43]. Automated clustering approaches, such as FlowSOM, were developed to overcome these limitations and have shown superior performance for large datasets compared to other algorithms [42, 43]. In this context, biological populations can be distinguished based on autofluorescence, with pollen exhibiting stronger signals than bacteria and fungi due to specific pigments. Building on that, this study presents an automated methodology for clustering LNA and HNA populations in atmospheric samples measured with FCM. In parallel with the detection of the LNA and HNA populations using Syto13, PI co-staining was used to identify dead and intact bioaerosols after clustering. This methodology is not restricted to atmospheric samples and can be applied to different ecosystems. It eliminates the need for manual clustering, reduces the risk of human error and bias, improves reproducibility, facilitates cross-study comparison, and can handle large datasets.

The total number of bioaerosols in Payerne [(2.47 ± 3.35) × 10^4^ m^−3^] was lower than the ones reported using FCM in Atlanta, USA [(5.50 ± 5.10) × 10^5^ m^−3^] [2] and in Beijing, China (5.78 × 10^6^ m^−3^) [22]. These elevated concentrations in urban areas are consistent with the greater density and diversity of anthropogenic emission sources observed in megacities. Payerne, on the other hand, represents a semi-rural European environment characterized by limited urbanization and proximity to agricultural activities [44]. Following the initial study [45] describing the LNA and HNA populations in aquatic environments, it was hypothesized that the LNA population might consist partly of dead microorganisms due to its lower nucleic acid intensity compared to HNA. In our samples, the strong correlation between the LNA and dead populations (r_s_ = 0.89, *P* < .001), together with microscopy images showing distorted bioaerosols as a sign of cellular death [45], further supported this hypothesis (Fig. S2D).

The contribution of bioaerosols to the PM_10_ mass was estimated based on the average particle size measured by FCM and assuming a mean density of 1.1 g cm^−3^ per particle [46]. The total bioaerosols contributed to 5.3 ± 5.0% in PM_10_ mass (Table S3) and by considering as organic matter (OM) the 30.8% of the total PM_10_ mass for the area [47] bioaerosols contributed for 17.7 ± 26.5% to OM (Table S3), which is ~2.3 times less than previous measurements in Payerne in 2012–2013, where primary biological material reached up to 41% of the total OM mass (Table S3) [46]. The lower contribution of bioaerosols to OM may reflect an increased proportion of inorganic and secondary aerosols in PM_10_ during the sampling period, which was reported to be dominant in Payerne [46, 47]. Within this chemically dominated background, the absence of correlations between the HNA population and PMs suggests that HNA is not directly associated with the sources of PMs.

FLEXPART simulations indicated a stronger influence of local air masses during the sampling period, supporting the hypothesis that the HNA population is primarily driven by local emission sources. In contrast, the strong correlations of LNA with PMs suggested either long-range transport (e.g. Saharan dust event on 8 April and 20 June, Fig. S3) or local emission sources, such as nearby agricultural activities mixed with long-range transport from the East or the West part of Europe (e.g. 1 May and 31 July, respectively, Fig. S3). This is further supported by the sampling period, which coincides with the sowing and harvesting season, known to increase the resuspension of soil-derived bioaerosols. This likely explains the presence of soil-associated taxa such as *Actinomycetota* and *Nitrososphaerota*, found in the LNA population [1, 3, 48].

Although fungi and protists appeared more abundant by one order of magnitude than bacteria based on qPCR data, differences in rRNA genes copy numbers (1–15 copies for prokaryotes versus 15–100 copies for eukaryotes) limit direct comparisons of kingdom abundance [49, 50]. PERMANOVA analysis revealed a more complex community structure than previously described [2]. Specifically, Spearman correlations indicated that the LNA population was mainly formed by the archaeal phylum *Nitrososphaerota* and the bacterial phylum *Actinomycetota* (r_s_ = 0.48 and 0.40, respectively, *P* < .10), which explains its small average cell size [1, 3]. While the HNA population had more taxa affiliated to the fungal classes of *Pichiomycetes* and *Ustilaginomycetes* (r_s_ = 0.40 and 0.49, respectively, *P* < .10), consistent with the observed bimodal size distribution (Fig. S4A). On the other hand, the pollen population was composed of several potential allergenic pollen, such as Fagales, Lamiales, Ranunculales, and Proteales (Fig. 6C), that could affect human health [51]. Pollen bursting generates SPPs, which likely contribute to the LNA population composition; however, as these particles contain little or no DNA, they are not captured by metagenomic analysis (Fig. S1E–H). In addition to these compositional differences between populations, significant seasonal variation (r__PERMANOVA__ = 0.19, *P* < .10) in bioaerosol composition was observed between spring and summer, with a high contribution from bacteria, fungi, and plants (r__PERMANOVA__ = 0.26, 0.23, 0.14, respectively, *P* < .10), aligning with previous studies reporting seasonal shifts [52–56].

The strong correlations between the various qPCR results highlighted the existence of complex, intricate interactions between kingdoms, further supported by Mantel and PERMANOVA analysis (Table S7). Such interactions include bacterial contributions to pollen germination and bursting (e.g. *Anabaena spp., Calothrix spp.*, or *Cylindrospermum spp.* belonging to the *Cyanobacteriota* phylum) [57], as well as bacterial–fungal and archaea–protist associations involved in key metabolic functions such as nitrogen fixation, photosynthesis, and organic matter degradation (e.g. *Bacillus spp.* with *Aspergillus spp., Diplonema* with *Holosporaceae*, or *Ciliates* with methanogenic archaea) [58–63]. These associations were also supported by microscopy observations, suggesting that they can persist during aerosolization without structural disruption (Fig. S2D). This level of complexity was also described in cloud environments [64, 65] and may contribute to the increased fraction of HNA due to the co-detection of nucleic acids from the symbionts and the host cells.

Source apportionment was carried out through factor analysis, integrating Spearman correlations among the obtained metagenomic data variables, meteorological parameters, and other atmospheric measurements. Seven main factors explained 72% of the variance: plant species, temperature, solar radiation flux, fungi, Dead-LNA (both populations), archaea, bacteria, and protists (Fig. S9A). The results showed that the principal sources of bioaerosols are from plants (*Caryophyllales* and *Asterales*) and fungi (*Malasseziomycetes* and *Tremellomycetes*), with factors accounting for 24% of the dataset. Solar radiation likely modulates bioaerosol abundance by promoting pollen release under elevated irradiance conditions [39]. The Dead–LNA associated factor, dominated by bacteria from *Bacillota, Actinomycetota, Bacteroidota*, and *Acidobacteriota*, accounted for 22%. These Phyla are frequently reported in the atmosphere [1]. The archaea (*Thermoplasmatota* and *Nanobdellota*) and protists (*Apicomplexa*) factors together explained 17% of the variability. The “complex matrix,” which accounted for 28% of the total variance, represented the dominant unresolved component in the factor analysis. Different sources of bioaerosols were detected and changed during the seasons, showing either a plant or an unidentified source in spring and shifting to a microbial contribution in summer, despite having a stable community composition (r__PERMANOVA__ = 0.05, *P* > .10) and diversity (Tables S7, S9, and  S10).

In conclusion, this study demonstrates that integrating wet cyclone sampling with FCM and metagenomic analysis allows the characterization and quantification of multiple bioaerosol populations, such as LNA, HNA, and pollen, and the discrimination between dead and intact cells, after DNA staining with a combination of nucleic acid dyes. Coupling FCM with metagenomic analysis, revealed that both LNA and HNA populations consisted of bacteria, archaea, and fungi, but with distinct compositions: LNA was mainly associated with the archaea *Nitrososphaerota* and bacteria *Actinomycetota*, whereas HNA was enriched by fungal classes such as *Pichiomycetes* and *Ustilaginomycetes*. These results highlight the complexity of atmospheric bioaerosols and their co-emission from soil and plant-related sources.

## Supplementary Material

ISMEC_SI_ycag167

## Data Availability

Experimental data are available at https://doi.org/10.5281/zenodo.17571642. FLEXPART results are available at https://atmo-access.nilu.no/PAYERN_HRDU.py.
