# Supplementary material for: Characterization of the atmospheric microbiome in a semi-rural area of Central Europe using flow cytometry
Source: ISME Commun. 2026 Jun 16;6(1):ycag167. doi: 10.1093/ismeco/ycag167 (PMC13540729; doi:10.1093/ismeco/ycag167)
Supplement: Supplementary file 1 [file ismec_si_ycag167.pdf]

Supplementary Information

Characterization of the Atmospheric Microbiome in a Semi-Rural Area of Central Europe Using Flow Cytometry

Ernest Abboud[1], Pierre Rossi[2], Benoit Crouzy[3], Nikolaos Evangeliou[4], Athanasios Nenes[1,5*], Kalliopi Violaki[1*]

[1]Laboratory of Atmospheric Processes and their Impacts, School of Architecture, Civil & Environmental Engineering, École Polytechnique Fédérale de Lausanne (EPFL), CH-1015 Lausanne, Switzerland

[2]Central Environmental Laboratory, School of Architecture, Civil & Environmental Engineering, École Polytechnique Fédérale de Lausanne, CH-1015 Lausanne, Switzerland

[3]Federal Office of Meteorology and Climatology MeteoSwiss, Chemin de l'Aérologie, CH-1530 Payerne, Switzerland

[4]Department for Atmospheric & Climate Research (ATMOS), Stiftelsen NILU (Former Norwegian Institute for Air Research), NO-2027 Kjeller, Norway

[5]Center for the Study of Air Quality and Climate Change (C-STACC), Institute of Chemical Engineering Sciences, Foundation for Research and Technology, GR-26504 Patras, Greece

[*]Corresponding authors:

Kalliopi Violaki, E-mail: ✉ kalliopi.violaki@epfl.ch, kviolaki@gmail.com

Athanasios Nenes, E-mail: ✉ athanasios.nenes@epfl.ch

S1. Supplementary Figures

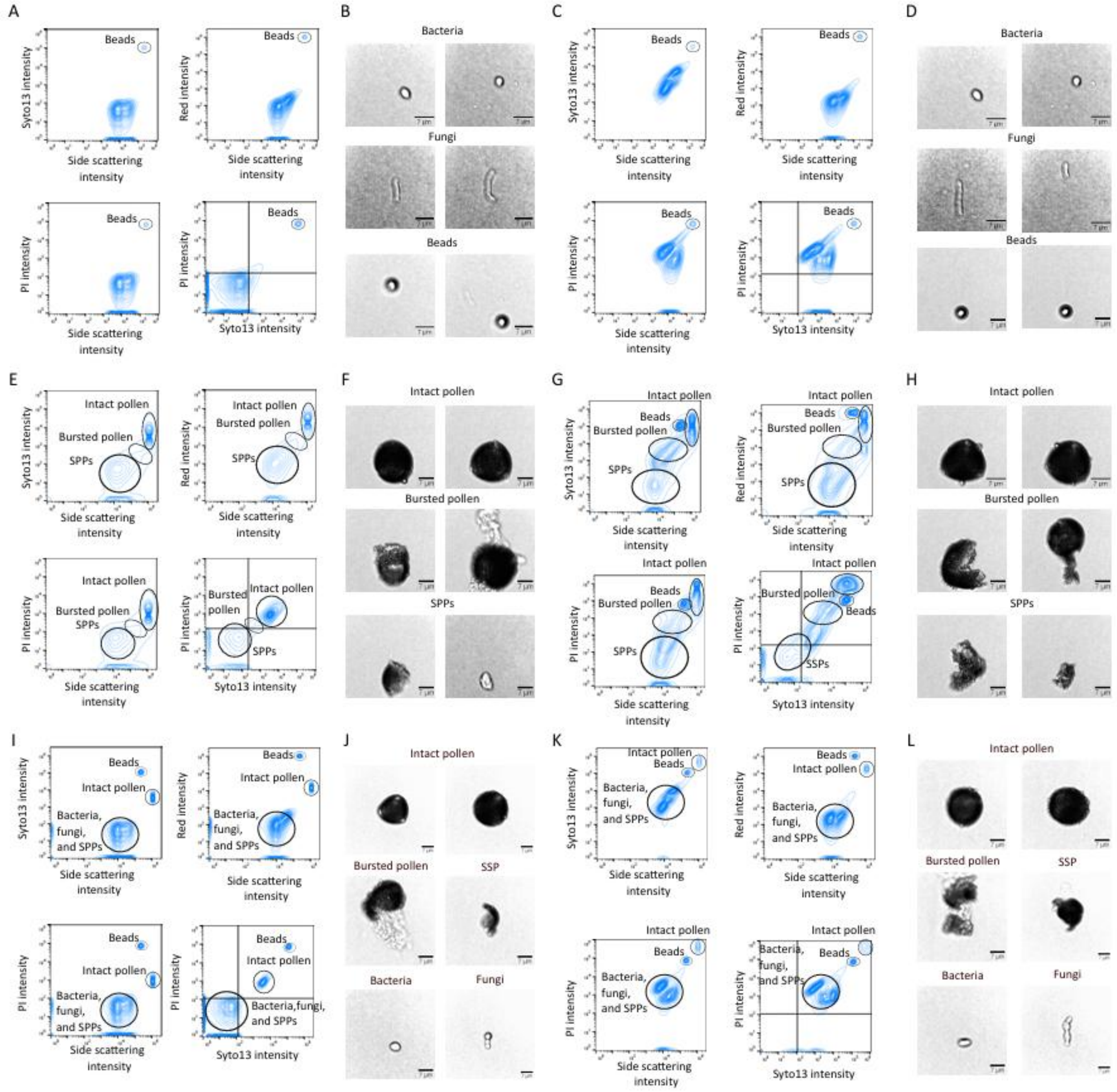

Figure S1: The Syto13 fluorescence intensity was detected in the BL1-A channel, the PI fluorescence intensity was detected in the YL2-A channel, and the pollen autofluorescence was detected in the RL1-A channel. **(A)** BL1-A vs. SSC-A, YL2-A vs. SSC-A, RL1-A vs. SSC-A, and YL2-A vs. BL1-A contour cytoplots (2%) of the commercial Zymo culture (containing 8 bacterial strains and 2 fungal strains, inactivated) before the addition of Syto13 and PI. Beads (internal control) are in the black circle. **(B)** Pictures of bacteria, fungi, and beads taken by the integrated FCM microscope. **(C)** Contour cytoplots (2%) of the commercial Zymo culture after the addition of Syto13 and PI were used to detect the increase of fluorescence in the BL1-A and YL2-A channels, with the following comparisons: BL1-A vs. SSC-A, YL2-A vs. SSC-A, RL1-A vs. SSC-A, and YL2-A vs. BL1-A. This confirmed that these microorganisms do not emit light in the RL1-A channel. **(D)** Pictures of bacteria, fungi, and beads taken by the flow cytometer integrated microscope. **(E)** Contour cytoplots (2%) of pollen (olive) autofluorescence: BL1-A vs. SSC-A, YL2-A vs. SSC-A, RL1-A vs. SSC-A, and YL2-A vs. BL1-A. **(F)** Pictures of intact, burst pollen and sub-pollen particles (SPPs). **(G)** Contour cytoplots (2%) showing the increase of

pollen fluorescence in the BL1-A, YL2-A, RL1-A, and SSC-A channels after the addition of Syto13 and PI: BL1-A vs. SSC-A, YL2-A vs. SSC-A, RL1-A vs. SSC-A, and YL2-A vs. BL1-A. **(H)** Pictures of intact, burst pollen and SPPs. **(I)** Contour cytoplots (2%) of the commercial Zymo culture and the olive pollen (representative of the local ash pollen): BL1-A vs. SSC-A, YL2-A vs. SSC-A, RL1-A vs. SSC-A, and YL2-A vs. BL1-A. **(J)** Pictures of intact, burst pollen, SPPs, bacteria, and fungi. **(K)** Contour cytoplots (2%) after the addition of Syto13 and PI: BL1-A vs. SSC-A, YL2-A vs. SSC-A, RL1-A vs. SSC-A, and YL2-A vs. BL1-A. There is no overlap between the different clusters identified. **(L)** Pictures of intact, burst pollen and SPPs confirming the composition of the different clusters.

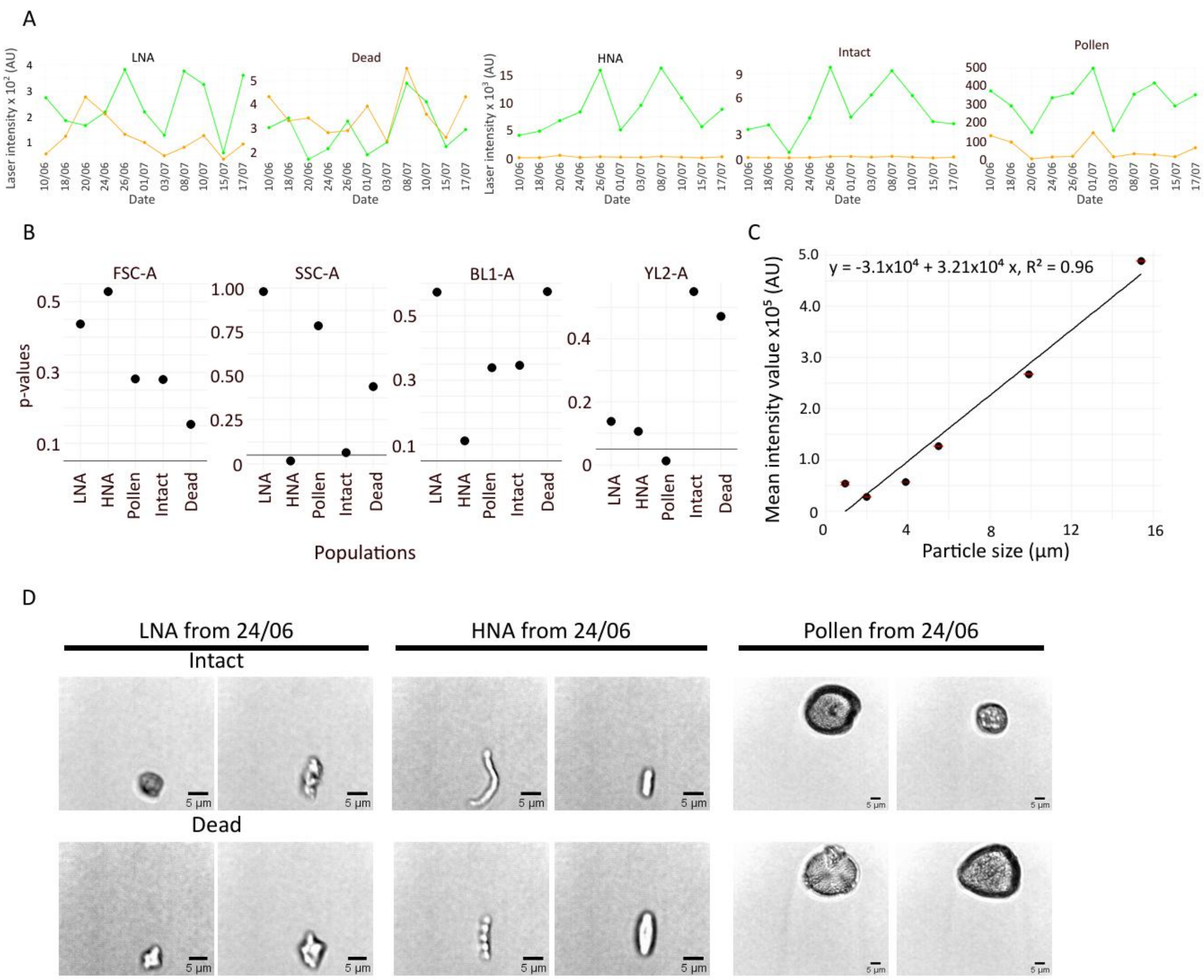

Figure S2: (A) Fluctuations in the geometric mean of the laser intensity of BL1-A (Syto13; green) and YL2-A (PI; yellow) (in arbitrary units, AU), as used to identify the LNA, HNA, pollen, dead, and intact populations, over 10 consecutive samples (sampled from 10 June 2024 to 17 July 2024). **(B)** p-values of the Shapiro-Wilk test to test the normal distribution of a small sample size (n=10) between the different lasers and the populations. **(C)** Linear regression was used to determine the size of individual particles (FCS-A vs. particle size in µm). **(D)** Example of pictures of bioaerosols found in the different populations (e.g. sample collected 24 June 2024; 24/06). The dead populations display a clear sign of cellular distortion; a phenotype found in apoptotic or necrotic cells [1].

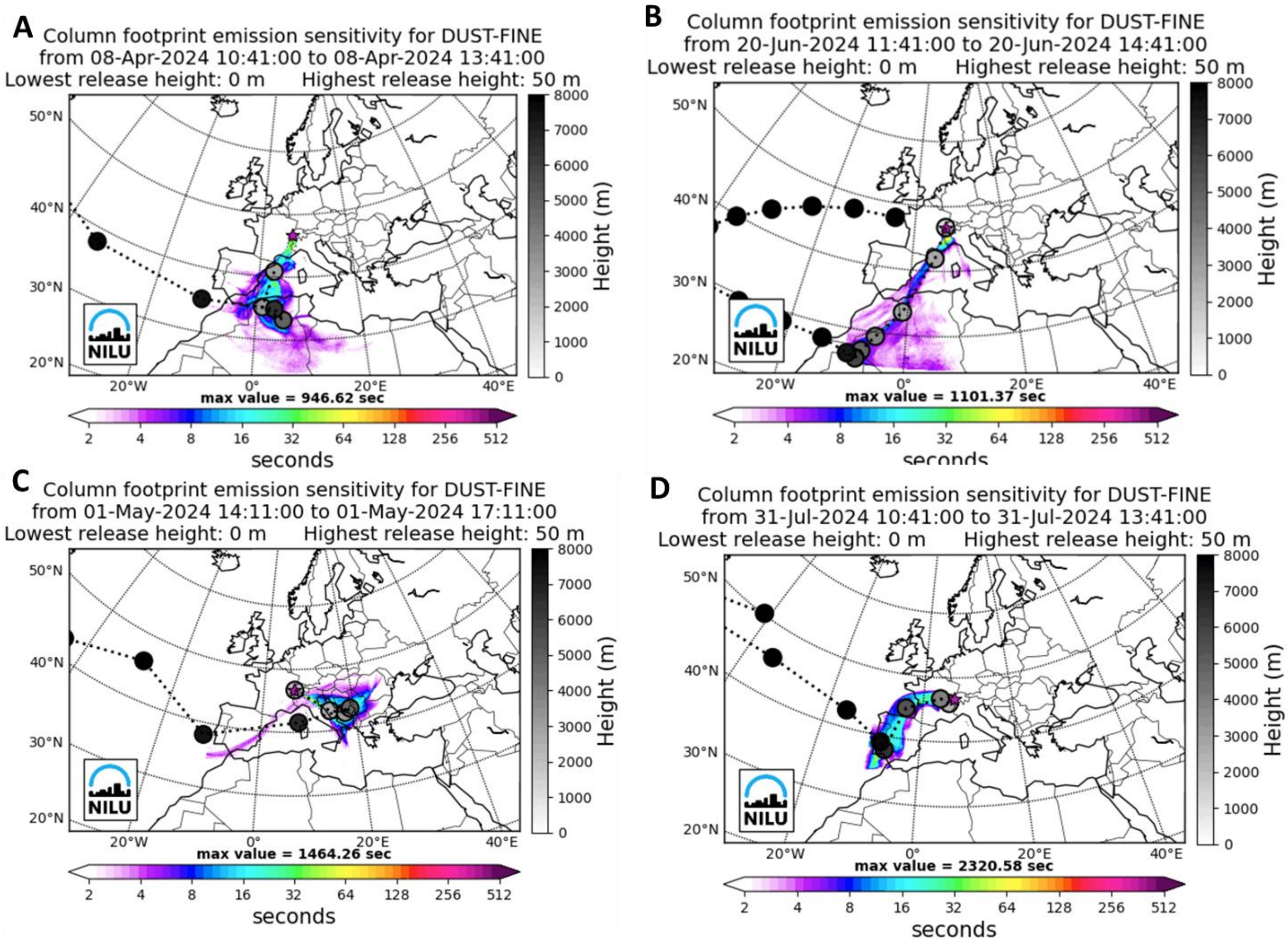

Figure S3: FLEXPART column footprint emission sensitivity for DUST-FINE particles for the Saharan dust events recorded during the sampling period: **(A)** on 8 April 2024 and **(B)** on 20 June 2024. The events on **(C)** 1 May 2024 and **(D)** 31 July 2024 are a typical mixture of long-range transport and local sources. The colour scale represents source–receptor sensitivity (residence time), with higher values indicating stronger contributions from potential source regions. The star denotes the receptor location, while the color indicates the spatial distribution of contributing air masses. The release height ranged from 0 to 50 m above ground level.

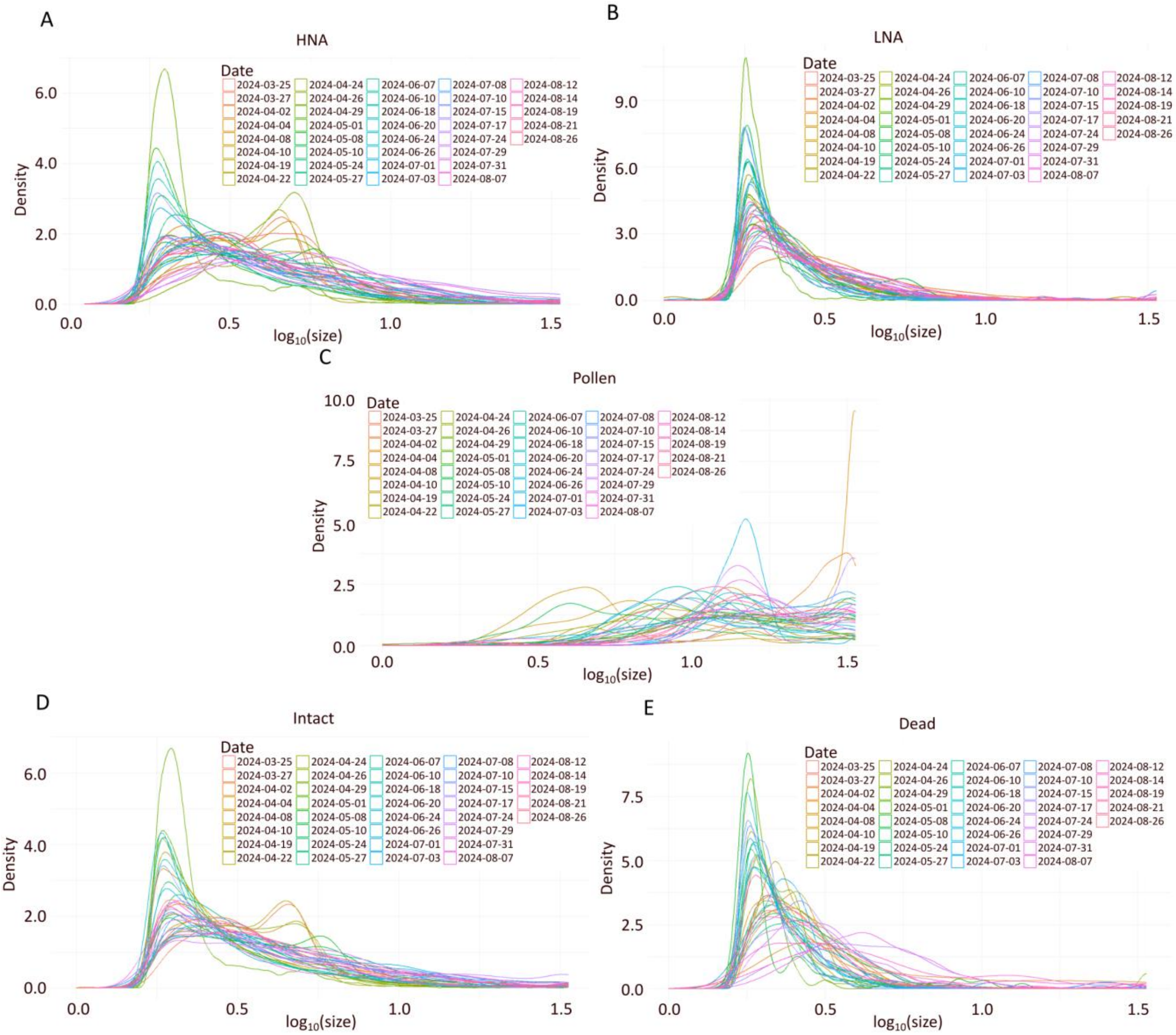

Figure S4: Size distribution of the **(A)** HNA, **(B)** LNA, **(C)** pollen, **(D)** intact, **(E)** dead populations on a Log_{10} transformed axis of the size (µm) calculated by using the FSC-A intensity with the equation found in Fig. S2C**.**

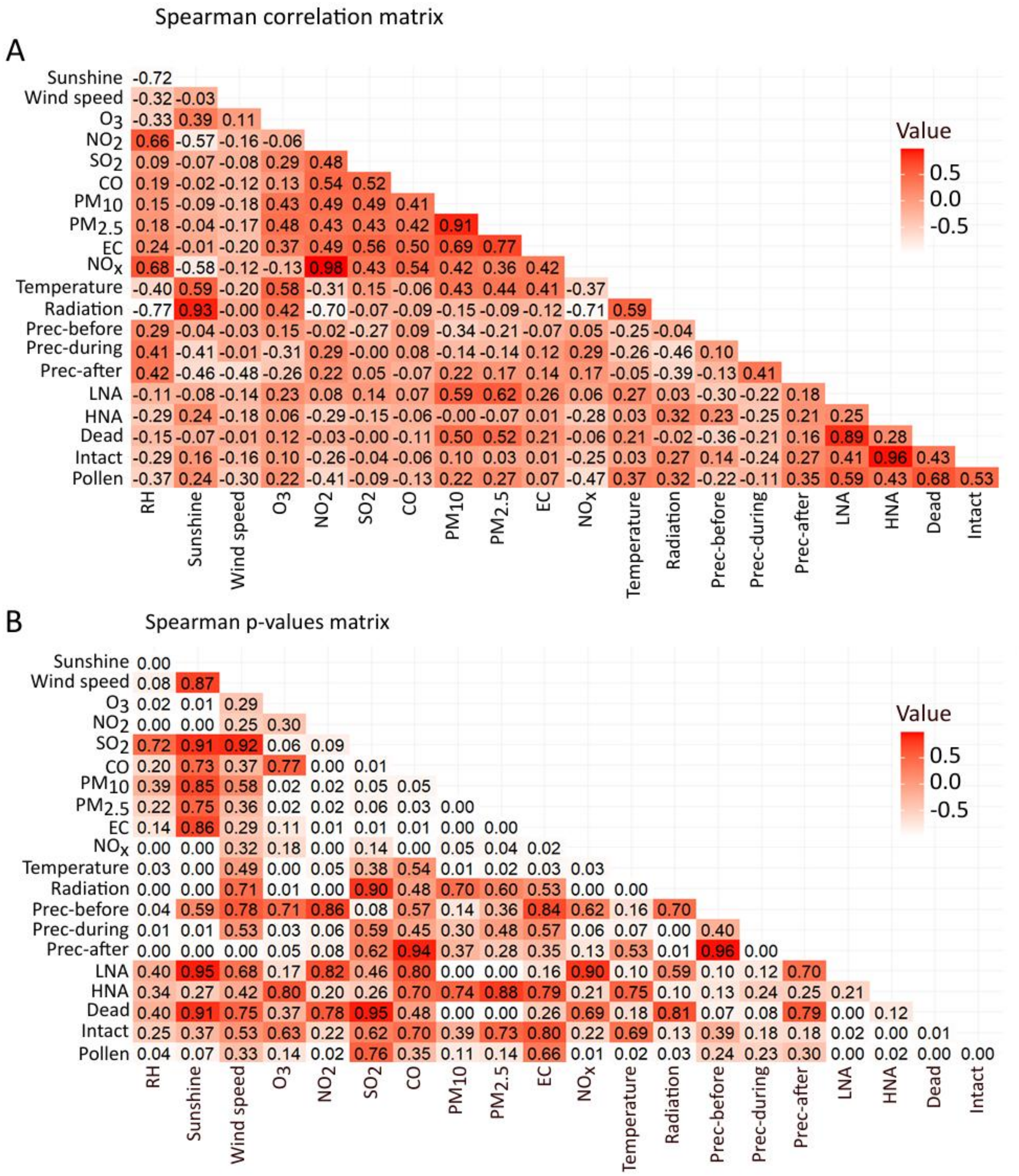

Figure S5: (A) Spearman correlation matrix, **(B)** p-values rounded to two decimals. Colour variations indicate that the closer to red, the closer to 1. “Prec-before” represents the precipitation before the sampling time; “Prec-during” represents the precipitation during the sampling time, and “Prec-after” represents the precipitation after the sampling time.

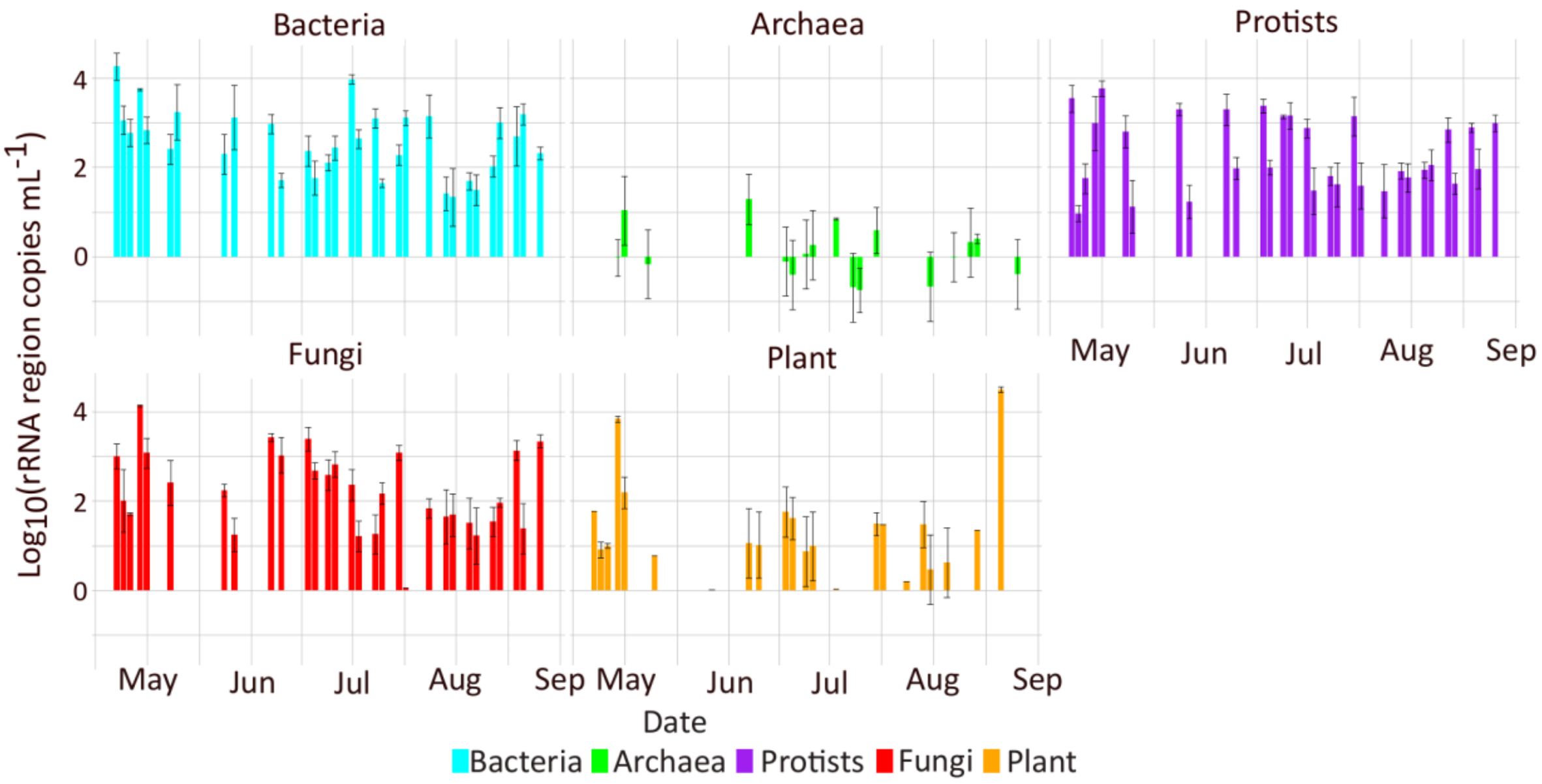

Figure S6: Quantification of the rRNA genes region by qPCR in the bacterial, archaeal, protist, fungal, and plant kingdoms, over the blank samples collected during the sampling period (25 March 2024 to 26 August 2024). Error bars show the standard deviation.

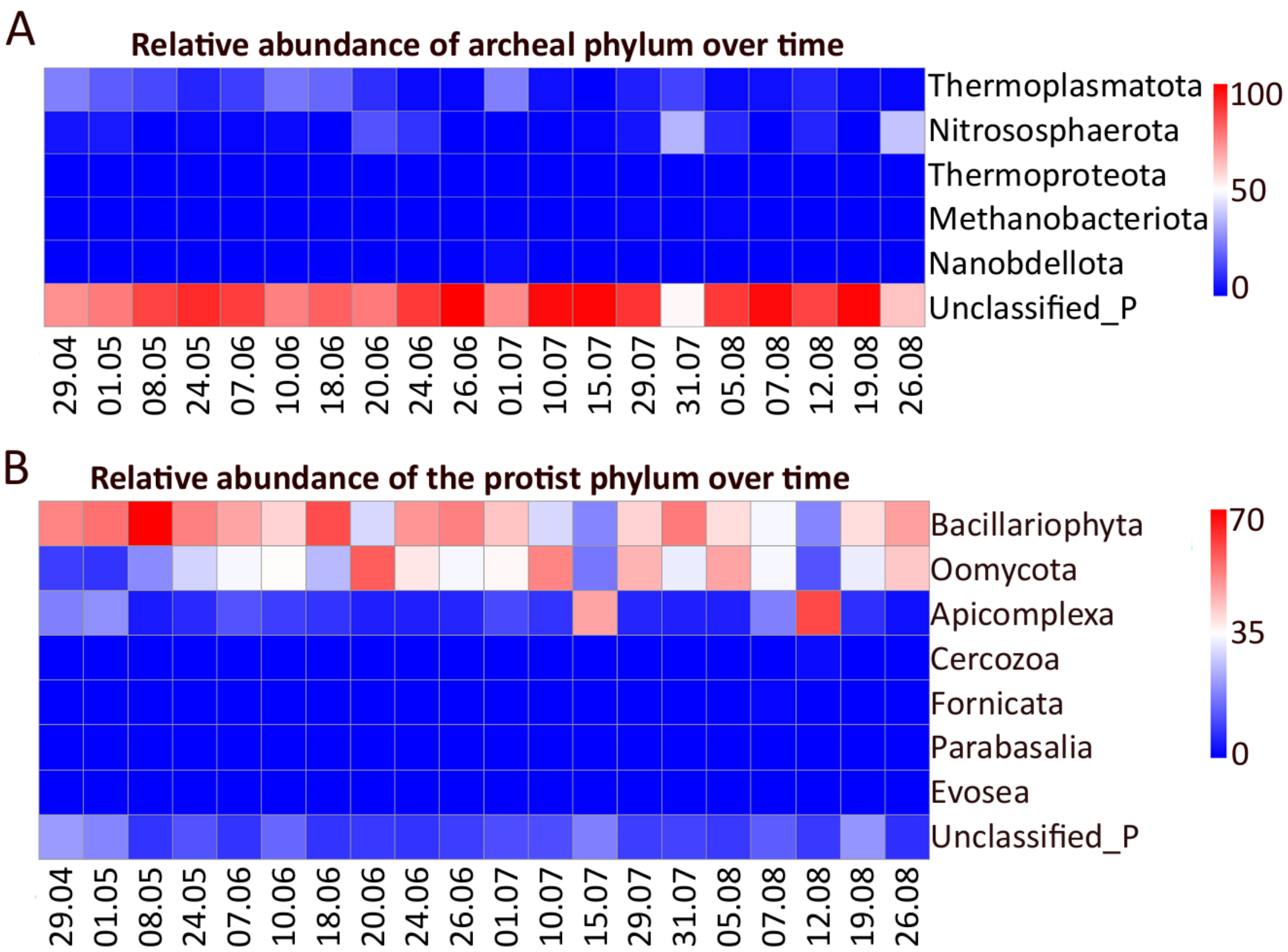

Figure S7: Taxonomic identification of a selection of 20 samples after Oxford Nanopore Technologies (ONT) sequencing at **(A)** the phylum level for archaea, **(B)** the phylum level of protists. Unclassified_P represents sequences unassigned at the phylum level.

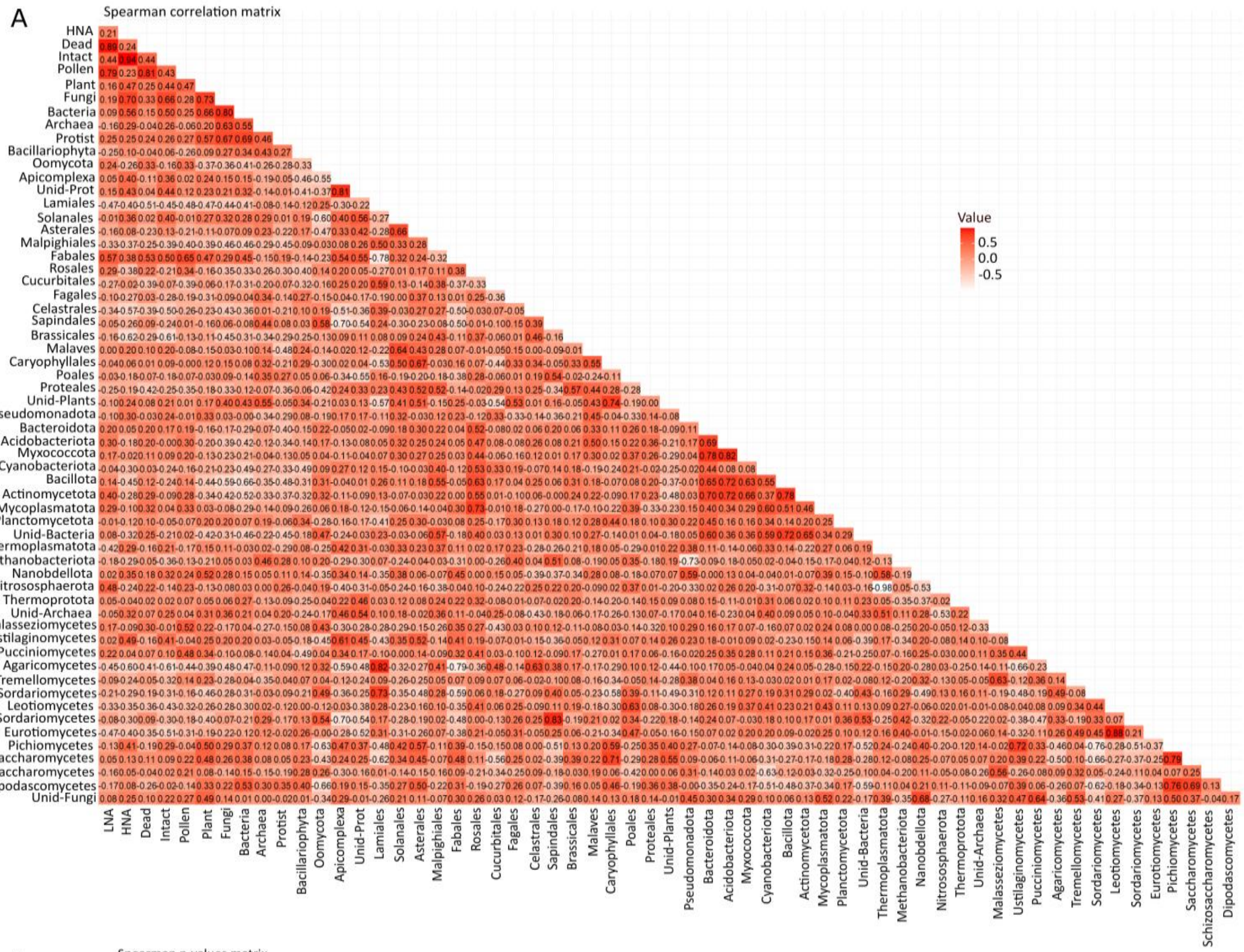

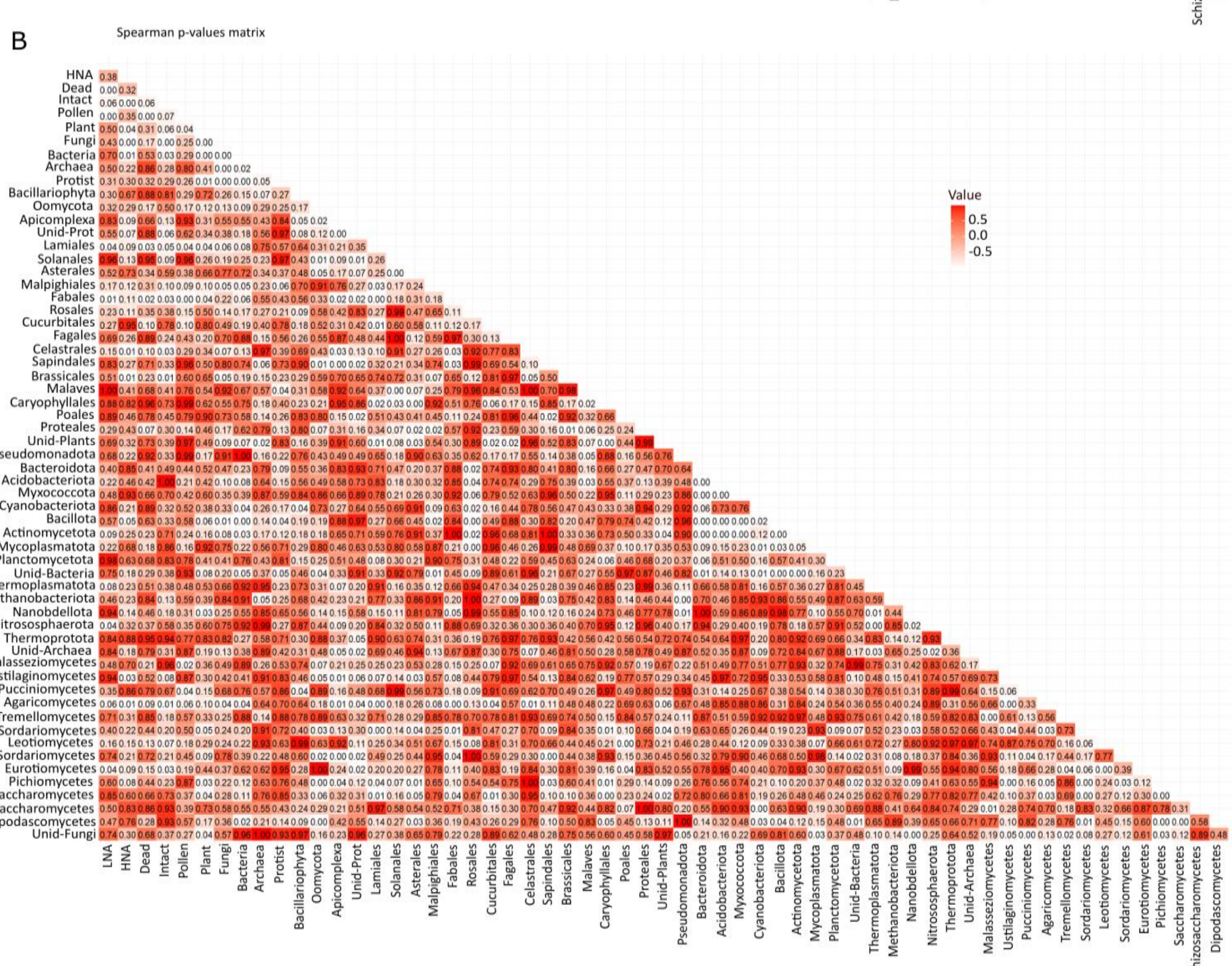

Figure S8: Spearman's rank correlation coefficient between the different FCM populations identified, qPCR amplicons, and the metagenomics analysis: **(A)** matrix, **(B)** p-values rounded to two decimal places. Colour variations indicate that the closer to red, the closer to 1. Unid stands for unidentified. Unid-Prot: unidentified protist. The phylum of bacteria, archaea, and protists, the class of fungi, and the order of plants are represented.

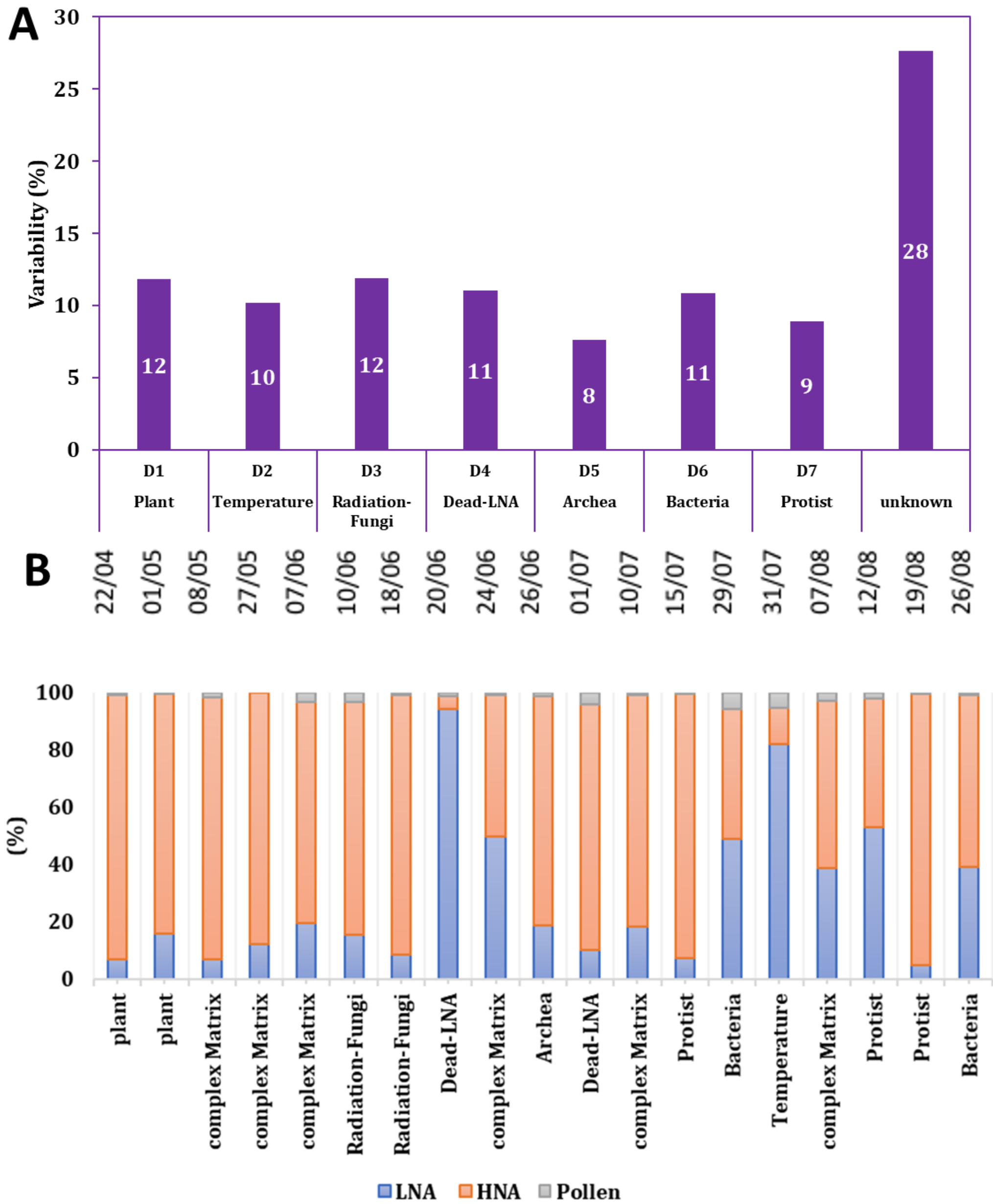

Figure S9: Factor analysis of bioaerosols composition and environmental variables **(A)** Factor loading of the seven main domains explaining 72% of the total variance. The x-axis shows the dominant variables that characterize each domain, and the y-axis shows the percentage contribution of each factor to the total data variability. **(B)** Factor analysis of bioaerosols on samples used for Oxford Nanopore Technologies (ONT) sequencing analysis (n = 19). The x-axis displays the dominant source of bioaerosols in each sample. The complex matrix is an unknown variable. The date of the samples is displayed at the top of every bar.

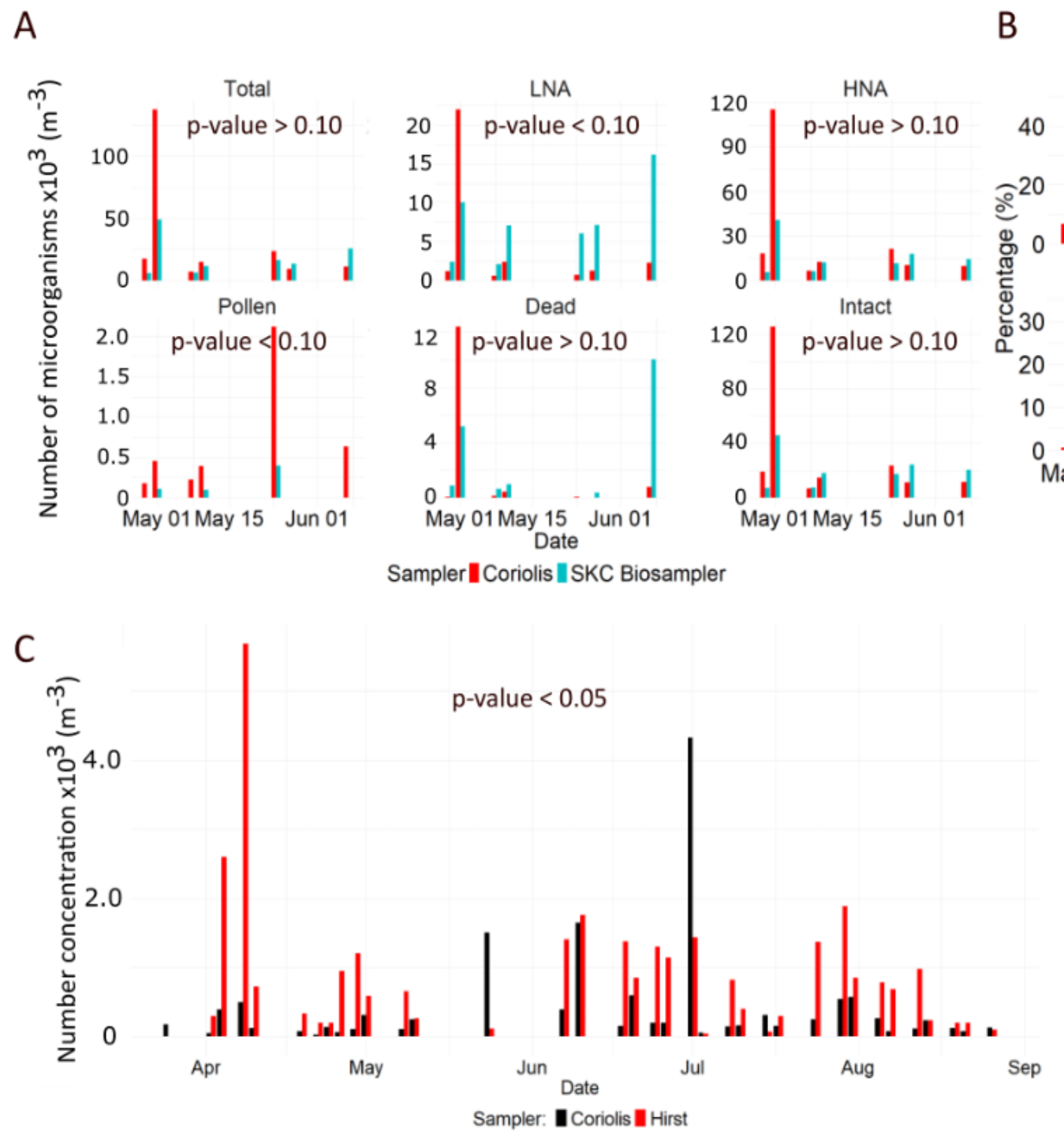

Figure S10: Comparison of **(A)** the number of bioparticles quantified (m^{-3}) and **(B)** their respective contribution (%) to the different populations in the Coriolis µ and SKC BioSampler. **(C)** comparison of the number of pollen particles quantified between Coriolis µ and the Hirst (m^{-3}).

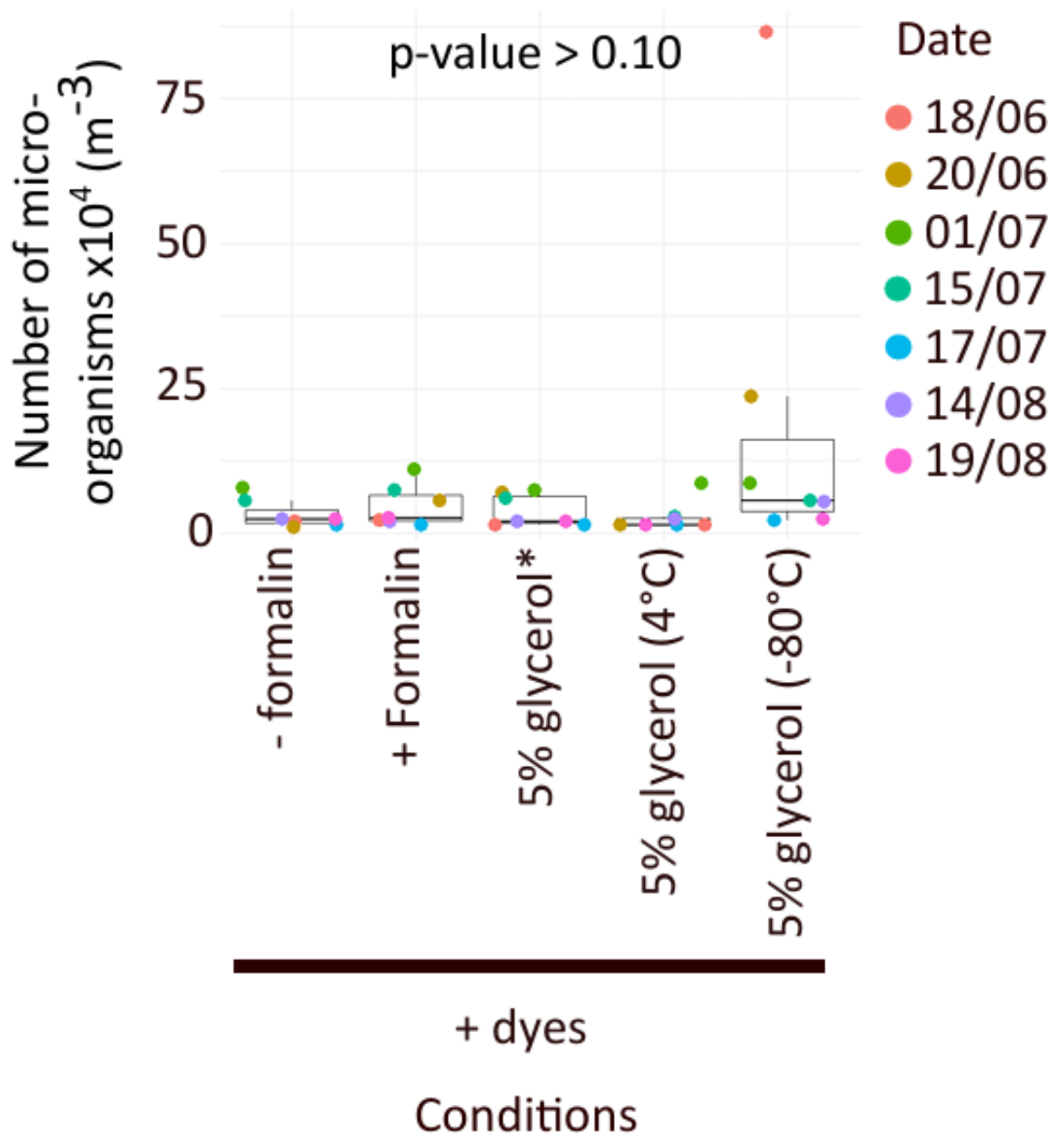

Figure S11: Comparison of the total number of bioparticles quantified in relation to the different storage techniques used. * indicates that these samples were analysed directly after the addition of 5% of glycerol.

S2. Supplementary Tables

Table S1: Number of clusters obtained for the three different algorithms tested: PhenoGraph, XShift, and FlowSOM on all samples collected during the campaign from 25 March 2024 to 26 August 2024 on FlowJo (dates are written as dd/mm/yyyy). Dimensions used for all clustering algorithms are: FSC-A, SSC-A, BL1-A, YL2-A, and RL1-A. Time of computation needed to generate output clusters was annotated as follows: A: below 5 minutes, B: 5-10 minutes, C: 10-15 minutes, D: 15-20 minutes, and E: above 20 minutes. *: crashed after 15 minutes of computation for Phenograph and did not work with Xshift. **: crashed after 12 minutes of computation for Phenograph. ***: crashed after 10 minutes of computation for Phenograph.

Date	Number of events (#)	Dimension	Phenograph clusters	k means by default	Time	Xshift clusters euclidien	k means by default	Time	FlowSOM clusters entered	Time
25/3/2024	51 031	6	17	158	B	19	280	A	40	A
27/3/2024	36 506	6	17	149	A	23	280	A	40	A
2/4/2024	17 494	6	20	129	A	14	280	A	40	A
4/4/2024	56 784	6	25	161	B	20	280	A	40	A
8/4/2024	212 957	6	*	197		*	280		40	A
10/4/2024	37 630	6	17	150	A	15	280	A	40	A
19/4/2024	18 419	6	13	131	A	7	280	A	40	A
22/4/2024	29 983	6	19	144	A	11	280	A	40	A
24/4/2024	38 869	6	22	151	A	13	280	A	40	A
26/4/2024	14 560	6	15	124	A	5	280	A	40	A
29/4/2024	34 440	6	20	148	A	19	280	A	40	A
1/5/2024	154 387	6	**	188		24	280	A	40	A
8/5/2024	8 861	6	14	111	A	7	280	A	40	A
10/5/2024	24 764	6	16	139	A	11	280	A	40	A
24/5/2024	36 350	6	18	149	A	12	280	A	40	A
27/5/2024	15 750	6	17	126	A	11	280	A	40	A
7/6/2024	18 135	6	16	130	A	8	280	A	40	A
10/6/2024	55 638	6	20	161	B	18	280	A	40	A
18/6/2024	27 232	6	18	141	A	9	280	A	40	A
20/6/2024	46 645	6	21	156	B	14	280	A	40	A
24/6/2024	42 477	6	18	139	B	19	280	A	40	A
26/6/2024	25 830	6	15	140	A	11	280	A	40	A
1/7/2024	84 710	6	***	172		22	280	A	40	A
3/7/2024	8 757	6	16	111	A	3	280	A	40	A
8/7/2024	17 953	6	16	130	A	6	280	A	40	A
10/7/2024	20 116	6	15	133	A	8	280	A	40	A
15/7/2024	65 340	6	21	165	C	24	280	A	40	A
17/7/2024	17 412	6	16	129	A	9	280	A	40	A
24/7/2024	9 559	6	15	113	A	5	280	A	40	A
29/7/2024	12 995	6	15	121	A	8	280	A	40	A
31/7/2024	38 978	6	18	151	B	11	280	A	40	A
5/8/2024	16 696	6	16	128	A	10	280	A	40	A
7/8/2024	7 746	6	13	107	A	7	280	A	40	A
12/8/2024	12 160	6	15	117	A	7	280	A	40	A

14/8/2024	20 267	6	16	133	A	10	280	A	40	A
19/8/2024	27 422	6	17	142	A	9	280	A	40	A
21/8/2024	11 849	6	15	119	A	8	280	A	40	A
26/8/2024	17 189	6	17	129	A	8	280	A	40	A

Table S2: Set of primers used for qPCR and ONT analysis for each of the targeted kingdoms with their respective annealing temperatures (°C).

Method	Targetted region	Targetted Kingdom	Primer name	Sequence	Annealing T° (°C)	Reference
qPCR	16S	Bacteria	338F	5′-ACTCCTACGGGAGGCAGCAG-3′	62	[2]
			520R	5′-ATTACCGCGGCTGCTGG-3′		
	16S	Archaea	931F	5′-AGGAATTGGCGGGGGAGCA-3′	60	[3]
			M1100R	5′-BGGGTCTCGCTCGTTRCC-3′		
	18S and ITS regions	Fungi	FungiQuant_F	5′-GGRAAACTCACCAGGTCCAG-3′	60	[4]
			FungiQuant_R	5′-GSWCTATCCCCAKCACGA-3′		
	18S and ITS regions	Protist	592F	5'-CGGTAATTCCAGCTCCARTAGC-3'	55	[5]
			1300R	5'-CACSAACTAARAACGGCCATGC-3'		
	18S and ITS regions	Plant	S2F	5'-ATGCGATACTTGGTGTGAAT-3'	58	[6]
			S3R	5'-GACGCTTCTCCAGACTACAAT-3'		
ONT	16S	Bacteria	8F	5'-AGAGTTTGATCMTGGCTCAG-3'	54	[7]
			1492R	5'-GGYTACCTTGTTACGACTT-3'		
	16S	Archaea	27F	5'-TTCCGGTTGATCCYGCCGGA-3'	56	[8]
			1492R	5'-GGYTACCTTGTTACGACTT-3'		
	18S and ITS regions	Fungi	Fw1	5′-TAGAGGAASTAAAAGTCGTAA-3′	50	[9]
			RGN3	5′-TCCTGAGGGAAACTTCG-3′		
	18S and ITS regions	Protist	592F	5'-CGGTAATTCCAGCTCCARTAGC-3'	55	[5]
			1300R	5'-CACSAACTAARAACGGCCATGC-3'		
	18S and ITS regions	Plant	ITS-u1	5′-GGAAGKARAAGTCGTAACAAGG-3′	52	[10]
			ITS-u4	5′-RGTTTCTTTTCCTCCGCTTA-3′		

Table S3: Size (µm) and percentage (%) contribution of each population to the average mass of OM (µg m^{-3}), with the standard deviation represented after the sign ±, during the sampling period. The pollen mass corresponds to pollen particles with diameters below 10 µm (within the PM_{10}). The density of one bioaerosol was considered equal to 1.1 g cm^{-3} [11].

Population	Average mass of PM_{10} (µg m^{-3})	Theoretical percentage of OM in PM_{10} (%) [12]	Theoretical mass of OM in PM_{10} (µg m^{-3})	Average size (µm)	Theoretical mass of bioaerosols (µg m^{-3})	Contribution of bioaerosols to OM (%)
Total	10.9 ± 7.7	30.8	3.4 ± 2.4	4.3 ± 1.7	0.59 ± 0.63	17.7 ± 26.5
LNA	10.9 ± 7.7	30.8	3.4 ± 2.4	3.7 ± 0.3	0.09 ± 0.17	2.6 ± 7.0
HNA	10.9 ± 7.7	30.8	3.4 ± 2.4	4.6 ± 0.3	0.49 ± 0.57	14.5 ± 24.0
Pollen	10.9 ± 7.7	30.8	3.4 ± 2.4	7.3 ± 1.0	0.02 ± 0.03	0.5 ± 1.4

Table S4: The total number of pollen particles quantified by FCM and manually counted under a light microscope by MeteoSwiss after collection with Hirst (both in m^{-3}) during the sampling period from 25 March 2024 to 26 August 2024 and the sampling time (dates are written as dd/mm/yyyy). For clarity, only the names given by MeteoSwiss with counts greater than 0 are shown. During the campaign, several types of pollen monitored by MeteoSwiss could not be identified and are not shown. These include: Alder, the amaranth family, cedar, common box, elder, elm, ginkgo, hazel, hornbeam, horse chestnut, larch, lime tree, maize, maple, palm family, ragweed, rye, sweetgum, and yew.

Date	Pollen identified by FCM (m^{-3})	Pollen Identified by MeteoSwiss: name (Count (#))	Total pollen MeteoSwiss (m^{-3})
25/3/2024	238	NA	0
27/3/2024	0	NA	0
2/4/2024	242	Birch (131), Common ash (98), Daisy family (33), Poplar (33)	295
4/4/2024	595	Birch (2211), Common ash (358), Spruce (33)	2602
8/4/2024	726	Birch (3575), Common ash (1171), Cypress family (326), Miscellaneous (131), Mulberry family (33), Nettle Hemp family (33), Oak (131), Olive family (65), Plane (98), Poplar (66), Sedge Family (33), Spruce (33)	5695
10/4/2024	179	Beech (33), Birch (195), Crucifers (33), Fir (33), Oak (33), Pine (33), Plane (163), Spruce (98), Willow (98)	719
19/4/2024	101	Beech (33), Birch (33), Fir (33), Miscellaneous (33), Oak (163), Sorrel dock (33)	328
22/4/2024	28	Oak (196)	196
24/4/2024	205	Miscellaneous (33), Oak (98), Spruce (33), Walnut (33)	197
26/4/2024	120	Beech (33), Birch (33), Crucifers (130), Grasses (66), Oak (456), Sorrel dock (33), Spruce (163), Willow (33)	947
29/4/2024	181	Grasses (33), Hophornbeam (33), Oak (748), Sorrel dock (33), Spruce (358)	1205
1/5/2024	457	Buttercup family (33), Miscellaneous (98), Oak (358), Pine (33), Spruce (33), Walnut (33)	588

8/5/2024	230	Beech (33), Buttercup family (33), Crucifers (33), Miscellaneous (33), Oak (195), Pine (293), Walnut (33)	653
10/5/2024	393	Cypress family (33), Miscellaneous (33), Pine (164), Sorrel dock (33)	263
24/5/2024	2118	Cypress family (24), Grasses (66), Pine (24)	114
27/5/2024	656	NA	0
7/6/2024	637	Cypress family (33), Grasses (748), Miscellaneous (163), Nettle Hemp family (196), Oak (98), Olive family (33), Pine (131)	1402
10/6/2024	2513	Grasses (1235), Miscellaneous (98), Nettle Hemp family (293), Pine (33), Plantain (33), Sorrel dock (33), Spruce (33)	1758
18/6/2024	232	Cypress family (98), Grasses (432), Miscellaneous (130), Nettle Hemp family (561), Olive family (33), Pine (57), Plantain (33), Sedge family (33)	1377
20/6/2024	991	Grasses (131), Nettle Hemp family (619), Pine (33), Sweet chestnut (65)	848
24/6/2024	261	Cypress family (33), Grasses (553), Miscellaneous (163), Nettle Hemp family (489), Sweet chestnut (65)	1303
26/6/2024	277	Cypress family (33), Grasses (554), Nettle Hemp family (325), Plantain (33), Sorrel dock (33), Sweet chestnut (163)	1141
1/7/2024	7539	Grasses (294), Miscellaneous (131), Nettle Hemp family (650), Plantain (33), Rose family (196), Sweet chestnut (130)	1434
3/7/2024	75	Spruce (33)	33
8/7/2024	208	Grasses (359), Miscellaneous (66), Nettle Hemp family (358), Plantain (33)	816
10/7/2024	287	Grasses (33), Nettle Hemp family (164), Plantain (98), Rose family (33), Sweet chestnut (66)	394
15/7/2024	497	Nettle Hemp family (33), Plantain (33)	66
17/7/2024	241	Grasses (131), Nettle Hemp family (130), Plantain (33)	294
24/7/2024	356	Grasses (293), Miscellaneous (65), Nettle Hemp family (846), Plantain (98), Sorrel dock (33), Umbellifers (33)	1368
29/7/2024	830	Grasses (228), Miscellaneous (33), Mugwort (33), Nettle Hemp family (1496), Plantain (99)	1889
31/7/2024	800	Grasses (66), Miscellaneous (33), Nettle Hemp family (553), Plantain (196)	848
5/8/2024	406	Daisy family (33), Grasses (33), Miscellaneous (33), Nettle Hemp family (618), Plantain (33), Spruce (33)	783
7/8/2024	106	Mercury (33), Miscellaneous (33). Nettle Hemp family (488), Plantain (131)	685
12/8/2024	167	Grasses (65), Miscellaneous (33), Nettle Hemp family (878)	976
14/8/2024	357	Nettle Hemp family (113), Plantain (122)	235
19/8/2024	186	Nettle Hemp family (196)	196
21/8/2024	105	Mercury (33), Nettle Hemp family (131), Plantain (33)	197
26/8/2024	174	Nettle Hemp family (66), Plantain (33)	99

Table S5: The qPCR amplicon average for all the targeted kingdoms in blanks and samples, along with their standard deviation and coefficient of variation (%).

Type	Kingdom	Average (m^{-3})	SD	CV (%)
Sample (blank corrected)	Bacteria	$2.64x10^5$	$3.58x10^5$	135.62
	Archaea	$2.38x10^2$	$3.62x10^2$	152.38
	Protists	$3.24x10^6$	$6.49x10^6$	200.27
	Fungi	$6.45x10^6$	$1.40x10^7$	217.74
	Plant	$4.34x10^5$	$1.08x10^6$	248.05
Blank	Bacteria	$1.80x10^3$	$3.94x10^3$	218.22
	Archaea	2.71	6.25	230.42
	Protists	$2.94x10^2$	$1.35x10^3$	146.28
	Fungi	$1.02x10^3$	$2.52x10^3$	248.13
	Plant	$1.31x10^3$	$5.88x10^3$	450.10

Table S6: Mantel test results for pairwise comparisons of sequencing read abundances among taxonomic kingdoms identified by ONT. Kingdom 1 and kingdom 2 indicate the tested groups; r_M denotes the Mantel correlation coefficient; ns indicates non-significant results.

Kingdom 1	Kingdom 2	r_M	p value	Significance
Bacteria	Fungi	0,23	0,06	Significant
Bacteria	Plant	0,24	0,02	Significant
Bacteria	Archaea	0,13	0,18	ns
Bacteria	Protists	0,90	0,00	Significant
Fungi	Plant	0,15	0,10	ns
Fungi	Archaea	0,43	0,01	Significant

Table S7: PERMANOVA results for comparisons of sequencing read abundances among taxonomic group kingdoms identified by ONT and the different atmospheric variables used in this study, the FCM populations, and qPCR data. r_P represents the $r_{PERMANOVA}$ and p the p-values. * represents a p-value < 0.10. Seasons are spring (April and May, n = 4) and summer (June, July, and August, n = 16).

	**All reads**		**Bacteria**		**Fungi**		**Plant**		**Archaea**		**Protists**	
Variables	r_P	p	r_P	p	r_P	p	r_P	p	r_P	p	r_P	p
Season	0,19	0,02*	0,26	0,01*	0,23	0,03*	0,14	0,06*	0,08	0,40	0,16	0,10
RH	0,09	0,09*	0,08	0,20	0,13	0,04*	0,07	0,26	0,08	0,24	0,02	0,81
Sunshine	0,05	0,60	0,03	0,64	0,10	0,13	0,03	0,91	0,01	0,89	0,03	0,68
Wind speed	0,03	0,87	0,03	0,74	0,02	0,89	0,03	0,84	0,01	0,85	0,10	0,14
O3	0,05	0,54	0,05	0,42	0,10	0,11	0,03	0,90	0,00	0,92	0,05	0,42
NO2	0,13	0,03*	0,09	0,17	0,22	0,02*	0,10	0,11	0,03	0,70	0,12	0,12
SO2	0,08	0,12	0,03	0,62	0,11	0,06*	0,09	0,11	0,02	0,73	0,17	0,04*
CO	0,11	0,03*	0,06	0,36	0,11	0,06*	0,14	0,00*	0,05	0,49	0,17	0,02*
PM10	0,10	0,07*	0,05	0,46	0,13	0,05*	0,09	0,12	0,14	0,07*	0,02	0,73
PM2.5	0,06	0,33	0,05	0,42	0,04	0,62	0,07	0,23	0,10	0,16	0,02	0,79
EC	0,05	0,59	0,04	0,52	0,07	0,24	0,04	0,66	0,03	0,66	0,05	0,42
NOx	0,14	0,04*	0,09	0,16	0,23	0,01*	0,11	0,10	0,03	0,71	0,12	0,10
Temperature	0,08	0,12	0,12	0,11	0,09	0,13	0,06	0,33	0,01	0,89	0,07	0,31
Radiation	0,05	0,51	0,02	0,80	0,11	0,08*	0,03	0,82	0,01	0,83	0,06	0,34
Prec_before	0,03	0,90	0,01	0,91	0,03	0,71	0,03	0,69	0,04	0,47	0,01	0,87
Prec_during	0,02	0,99	0,01	0,96	0,02	0,96	0,03	0,72	0,07	0,29	0,03	0,66
Prec_after	0,03	0,91	0,02	0,90	0,05	0,50	0,03	0,89	0,02	0,80	0,02	0,77
LNA	0,11	0,04*	0,16	0,03*	0,06	0,39	0,09	0,13	0,27	0,00*	0,09	0,17
HNA	0,11	0,04*	0,15	0,05*	0,06	0,33	0,08	0,13	0,26	0,00*	0,09	0,18
Dead	0,08	0,12	0,11	0,11	0,08	0,15	0,06	0,28	0,11	0,14	0,11	0,12
Intact	0,08	0,11	0,11	0,13	0,08	0,15	0,06	0,28	0,11	0,14	0,11	0,12
Pollen	0,05	0,46	0,03	0,65	0,07	0,30	0,05	0,52	0,04	0,56	0,05	0,47
qPCR_bacteria	0,17	0,01*	0,22	0,01*	0,18	0,02*	0,13	0,04*	0,11	0,14	0,12	0,10
qPCR_archaea	0,11	0,04*	0,12	0,08*	0,19	0,02*	0,07	0,23	0,01	0,93	0,15	0,08*
qPCR_protists	0,09	0,08*	0,08	0,21	0,14	0,02*	0,07	0,26	0,02	0,73	0,16	0,04*
qPCR_fungi	0,13	0,03*	0,19	0,02*	0,19	0,02*	0,09	0,13	0,01	0,88	0,11	0,12
qPCR_plant	0,11	0,06*	0,08	0,21	0,08	0,19	0,20	0,01*	0,14	0,07*	0,01	0,90

Table S8: Percentage of classified and unclassified sequences after ONT sequence analysis for all the targeted kingdoms with their standard deviation.

**Kingdom**	**Classified sequences (%)**	**SD**	**Unclassified sequences (%)**	**SD**
Total	93.4	10.5	6.6	10.5
Bacteria	98.5	1.8	1.5	1.8
Archaea	18.9	13.1	81.1	13.1
Protists	98.5	2.4	1.5	2.4
Fungi	96.5	14.7	3.5	14.7
Plant	98.7	6.0	1.3	6.0

Table S9: Summary of alpha diversity metrics characterizing bioaerosol communities on samples, represented by the date, sent for ONT analysis. Dates are written as dd/mm/yyyy. Seasons are spring (April and May, n = 4) and summer (June, July, and August, n = 16).

Date	Season	Species richness S	Shannon H	Shannon diversity number	Inverse Simpson diversity number	Pielou's Evenness	Shannon Evenness	Simpson Evenness
29/4/2024	Spring	50	2,94	18,82	11,94	0,75	0,38	0,24
1/5/2024	Spring	47	2,75	15,69	10,44	0,72	0,33	0,22
8/5/2024	Spring	52	2,86	17,40	10,75	0,72	0,33	0,21
24/5/2024	Spring	48	2,85	17,21	11,01	0,74	0,36	0,23
7/6/2024	Summer	47	2,89	17,93	11,19	0,75	0,38	0,24
9/6/204	Summer	51	2,86	17,52	11,41	0,73	0,34	0,22
18/6/2024	Summer	52	2,96	19,24	12,10	0,75	0,37	0,23
20/6/2024	Summer	52	3,03	20,73	14,58	0,77	0,40	0,28
24/6/2024	Summer	52	2,90	18,10	12,01	0,73	0,35	0,23
26/6/2024	Summer	47	2,74	15,48	10,32	0,71	0,33	0,22
1/7/2024	Summer	44	2,67	14,38	10,06	0,70	0,33	0,23
10/7/2024	Summer	49	2,85	17,21	11,29	0,73	0,35	0,23
15/7/2024	Summer	52	2,96	19,23	12,28	0,75	0,37	0,24
29/7/2024	Summer	55	3,02	20,58	12,86	0,75	0,37	0,23
31/7/2024	Summer	52	3,07	21,61	15,50	0,78	0,42	0,30
7/8/2024	Summer	45	2,85	17,22	11,82	0,75	0,38	0,26
12/8/2024	Summer	51	2,91	18,42	11,95	0,74	0,36	0,23
19/8/2024	Summer	51	2,92	18,58	11,62	0,74	0,36	0,23
26/8/2024	Summer	47	2,81	16,59	11,71	0,73	0,35	0,25

Table S10: Normality assessment of alpha diversity indices using the Shapiro–Wilk test, followed by pairwise comparisons using parametric (t-test) and non-parametric (Wilcoxon) tests. Spring_p and Summer_p denote the Shapiro–Wilk p-values for spring and summer, respectively. In spring normal and summer normal columns, False indicates if p-value < 0.05 and True if p-value> 0.10, p-value columns show the p-value of the pairwise test used, and ns indicates non-significant results.

Index	Spring_p	Spring normal	Summer_p	Summer normal	Test used	p-value	Significant
Species richness	0,80	True	0,15	True	t-test	0,69	ns
Shannon H	0,82	True	0,98	True	t-test	0,39	ns
Shannon diversity number	0,83	True	0,99	True	t-test	0,35	ns
Inverse Simpson diversity number	0,53	True	0,04	False	Wilcoxon	0,20	ns
Pielou's Evenness	0,85	True	0,96	True	t-test	0,37	ns
Shannon Evenness	0,31	True	0,81	True	t-test	0,37	ns
Simpson Evenness	0,92	True	0,01	False	Wilcoxon	0,24	ns

Table S11: Comparison of the quantified total number of bioaerosols and the different populations by FCM between the Coriolis µ sampler and the SKC BioSampler.

Sampler used	Population	Average (m^{-3})	SD
Coriolis	Total	3.16E+04	4.71E+04
Coriolis	LNA	4.35E+03	7.83E+03
Coriolis	HNA	2.77E+04	3.90E+04
Coriolis	Pollen	5.74E+02	7.11E+02
Coriolis	Dead	2.00E+03	4.59E+03
Coriolis	Intact	3.05E+04	4.22E+04
SKC BioSampler	Total	1.84E+04	1.52E+04
SKC BioSampler	LNA	7.29E+03	4.83E+03
SKC BioSampler	HNA	1.55E+04	1.20E+04
SKC BioSampler	Pollen	8.74E+01	1.47E+02
SKC BioSampler	Dead	2.60E+03	3.72E+03
SKC BioSampler	Intact	2.03E+04	1.30E+04

Table S12: A comparison of the total number of quantified bioaerosols using different sample conservation methods. The final concentration of formalin was 0.74%. Dates are written as dd/mm/yyyy.

**Date**	**Conditions**	**Number (m^{-3})**
18/6/2024	- formalin	1.92E+04
18/6/2024	+ formalin	2.25E+04
18/6/2024	5% glycerol (direct analysis)	1.45E+04
18/6/2024	5% glycerol (4°C)	1.42E+04
18/6/2024	5% glycerol (-80°C)	8.65E+05
20/6/2024	- formalin	1.05E+04
20/6/2024	+ formalin	5.55E+04
20/6/2024	5% glycerol (direct analysis)	6.86E+04
20/6/2024	5% glycerol (4°C)	1.27E+04
20/6/2024	5% glycerol (-80°C)	2.36E+05
1/7/2024	- formalin	7.81E+04
1/7/2024	+ formalin	1.09E+05
1/7/2024	5% glycerol (direct analysis)	7.43E+04
1/7/2024	5% glycerol (4°C)	8.54E+04
1/7/2024	5% glycerol (-80°C)	8.61E+04
15/7/2024	- formalin	5.49E+04
15/7/2024	+ formalin	7.36E+04
15/7/2024	5% glycerol (direct analysis)	5.98E+04
15/7/2024	5% glycerol (4°C)	2.69E+04
15/7/2024	5% glycerol (-80°C)	5.56E+04
17/7/2024	- formalin	1.43E+04
17/7/2024	+ formalin	1.33E+04
17/7/2024	5% glycerol (direct analysis)	1.38E+04
17/7/2024	5% glycerol (4°C)	1.28E+04
17/7/2024	5% glycerol (-80°C)	2.21E+04

14/8/2024	- formalin	2.40E+04
14/8/2024	+ formalin	1.98E+04
14/8/2024	5% glycerol (direct analysis)	1.91E+04
14/8/2024	5% glycerol (4°C)	2.28E+04
14/8/2024	5% glycerol (-80°C)	5.34E+04
19/8/2024	- formalin	2.30E+04
19/8/2024	+ formalin	2.58E+04
19/8/2024	5% glycerol (direct analysis)	1.88E+04
19/8/2024	5% glycerol (4°C)	1.35E+04
19/8/2024	5% glycerol (-80°C)	2.33E+04

S3. Supplementary method

S3.1 Collection efficiency of Coriolis µ compared to the SKC BioSampler and Hirst devices

The collection efficiency of Coriolis µ, known in the literature, is limited. However, [13] demonstrated that the Coriolis δ (flow rate of 630 L min^{-1}) can efficiently collect particles up to 10 µm. The particle collection efficiency for particles of 1, 2.4, 4.6, and 10 µm was 49.1 ± 7.8%. 40.6 ± 6.8%. 83.8 ± 21.5%, and 91.6 ± 16.6%, respectively. They found no statistical difference in collection efficiency between the Coriolis δ and the Hirst for pollen [13, 14]. However, [15] found a statistical difference in the amount of pollen detected by doing the same comparison (Coriolis δ, flow rate of 200 L min^{-1}). In addition, the Coriolis µ (300 L min^{-1}) showed lower collection efficiency for fungal spores (33.5 ± 6.1 %) compared to the BioSampler (52.1 ± 3.6%), whereas its efficiency for bacteriophage *MS2*, *Bacillus atrophaeus* spores, and *Serratia marcescens* was comparable to or higher than that of the SKC BioSampler [16, 17]. The latest efficiency collection on the Coriolis µ indicated values of 7.0 ± 0.3%, 50.0 ± 2.0%, and 91.0 ± 11.0% for particles measuring 0.8, 1, and 3 µm, respectively, at a flow rate of 300 L min^{-1}. These values are in the same order of magnitude as those already reported [13, 18].

In our study, the SKC BioSampler was run in parallel with the Coriolis µ (n=7, flow rate = 12.5 L.min^{-1}). The SKC BioSampler was chosen since its efficiency for particles smaller than 5 µm is well studied [19]. In addition, the pollen cluster identified by FlowSOM was compared to the manual counting of pollen from the Hirst (data given by MeteoSwiss). The total quantification of bioaerosols showed that in 71% of the samples, Coriolis µ had a higher collection of bioaerosols (Fig. S10A, Table S11). In 85% of the samples collected by the SKC BioSampler, the number of bioaerosols in the LNA population is higher, and only 15% of the samples collected by the SKC BioSampler showed a higher collection for the HNA population. The enhanced sampling of LNA populations can be attributed to their smaller particle size compared to HNA populations and the better collection efficiency of the SKC BioSampler for these smaller particles (Fig. S10A and B). The higher number of dead cells was collected using the SKC BioSampler (71% of samples). However, the effect of sampling conditions on cell viability remains uncertain. Its lower flow rate (27 times lower than Coriolis µ), likely reduces mechanical stress, while the higher flow rate of Coriolis µ may instead promote the disruption of already weakened cells. The absence of significant differences between the two instruments indicates that these variations do not impact the overall or specific quantification

of populations in our study. On the contrary, pollen is the only population that showed a significant difference ($p < 0.10$) between the two samplers (Fig. S10A). Moreover, pollen showed a significant difference ($p < 0.10$) compared to the Hirst quantification (Fig. S10C). It indicated that although the Coriolis µ is more efficient at collecting larger bioparticles than the SKC BioSampler, it remains less efficient than the Hirst when bioparticles reach the size of pollen, which is consistent with the findings of [15]. It is important to note that pollen quantification using FlowSOM included only intact pollen, and this number may be underestimated due to pollen bursting upon contact with water. In addition, the Hirst has known limitations. Several studies have shown that their representativeness and accuracy are reduced compared to automated pollen monitors, due to manual counting errors, limited temporal resolution, averaging effects, and flow rate measurements [20, 21].

It is worth noting that due to the low collection efficiency of Coriolis µ for small particles, coupled with the 3h sampling duration, the possibility of small particle re-aerosolization through the blower exhaust or coagulation inside the cone cannot be ruled out. Future work is required to address this possible issue [22]. [23] reported a collection efficiency of 34 ± 10% for living and 30 ± 5% for dead (by heat) *Escherichia coli* using the Coriolis µ, suggesting similar sampling efficiencies. To overcome the uncertainties arising from the low collection efficiency of high-volume wet cyclones, [24] corrected the HNA cell counts (sampled with the SpinCon II) using ABC-type WIBS measurements of particles between 1 and 5 µm, based on the observed correlation between the two datasets. Also, the high flow rate of these instruments may induce an extracellular stress on the bioaerosols, affecting their distribution in the LNA and HNA populations, as well as their viability and integrity by potentially destroying the already fragile bioaerosols (Fig. S10A and B).

S3.2 Storage protocol of the samples

Formalin is a toxic compound that can be excited at a wavelength of 558 nm and emit light at a wavelength of 583 nm. To investigate the potential use of alternative preservation methods, a set of samples (n=7) was aliquoted and stored with 5% glycerol at either 4°C or -80°C. Both sets of samples were analysed after two weeks. This test also aimed to evaluate the feasibility of quantifying bioaerosols after freezing with glycerol, as it is routinely used as a cryoprotectant to freeze and conserve cellular culture [25].

After FCM quantification, the Kruskal-Wallis p-value ($p > 0.10$) indicated no statistically significant change in the quantification of the bioaerosols (Fig. S11). However, the increase in the number of bioaerosols by 61-fold and 18-fold, respectively, after sampling on 18/06 and 20/06, and subsequent storage at -80°C, indicated that freezing and thawing cycles cannot be applied to bioaerosol samples. This is because the freezing process caused the samples to break into smaller particles, even with the addition of a cryoprotectant (Fig. S11 and Table S12). This observation led us to use formalin for the fixation of the samples.

S4. Supplementary References

1. Galluzzi L, Vitale I, Aaronson SA, Abrams JM, Adam D, Agostinis P, et al. Molecular mechanisms of cell death: Recommendations of the Nomenclature Committee on Cell Death 2018. *Cell Death Differ* 2018;**25**. https://doi.org/10.1038/s41418-017-0012-4

2. Yu Y, Lee C, Kim J, Hwang S. Group-specific primer and probe sets to detect methanogenic communities using quantitative real-time polymerase chain reaction. *Biotechnol Bioeng* 2005;**89**. https://doi.org/10.1002/bit.20347

3. Einen J, Thorseth IH, Øvreås L. Enumeration of Archaea and Bacteria in seafloor basalt using real-time quantitative PCR and fluorescence microscopy. *FEMS Microbiol Lett* 2008;**282**. https://doi.org/10.1111/j.1574-6968.2008.01119.x

4. Liu CM, Kachur S, Dwan MG, Abraham AG, Aziz M, Hsueh PR, et al. FungiQuant: a broad-coverage fungal quantitative real-time PCR assay. *BMC Microbiol* 2012;**12**. https://doi.org/10.1186/1471-2180-12-255

5. Venter PC, Nitsche F, Domonell A, Heger P, Arndt H. The Protistan Microbiome of Grassland Soil: Diversity in the Mesoscale. *Protist* 2017;**168**. https://doi.org/10.1016/j.protis.2017.03.005

6. Chen S, Yao H, Han J, Liu C, Song J, Shi L, et al. Validation of the ITS2 region as a novel DNA barcode for identifying medicinal plant species. *PLoS One* 2010;**5**. https://doi.org/10.1371/journal.pone.0008613

7. Weisburg WG, Barns SM, Pelletier DA, Lane DJ. 16S ribosomal DNA amplification for phylogenetic study. *J Bacteriol* 1991;**173**. https://doi.org/10.1128/jb.173.2.697-703.1991

8. DeLong EF. Archaea in coastal marine environments. *Proc Natl Acad Sci U S A* 1992;**89**. https://doi.org/10.1073/pnas.89.12.5685

9. Ohta A, Nishi K, Hirota K, Matsuo Y. Using nanopore sequencing to identify fungi from clinical samples with high phylogenetic resolution. *Sci Rep* 2023;**13**. https://doi.org/10.1038/s41598-023-37016-0

10. Cheng T, Xu C, Lei L, Li C, Zhang Y, Zhou S. Barcoding the kingdom Plantae: New PCR primers for ITS regions of plants with improved universality and specificity. *Mol Ecol Resour* 2016;**16**. https://doi.org/10.1111/1755-0998.12438

11. Marcovecchio F, Perrino C. Bioaerosol contribution to atmospheric particulate matter in indoor university environments. *Sustainability (Switzerland)* 2021;**13**. https://doi.org/10.3390/su13031149

12. Hüglin C, Grange SK. Chemical characterisation and source identification of PM10 and PM2.5 in Switzerland. *Claudia Zellweger-Fäsi* . David Jaramillo Vogel, 2021.

13. Carvalho E, Sindt C, Verdier A, Galan C, O'Donoghue L, Parks S, et al. Performance of the Coriolis air sampler, a high-volume aerosol-collection system for quantification of

airborne spores and pollen grains. *Aerobiologia (Bologna)* 2008;**24**. https://doi.org/10.1007/s10453-008-9098-y

14. Hirst JM. AN AUTOMATIC VOLUMETRIC SPORE TRAP. *Annals of Applied Biology* 1952;**39**. https://doi.org/10.1111/j.1744-7348.1952.tb00904.x

15. Gómez-Domenech M, García-Mozo H, Alcázar P, Brandao R, Caeiro E, Munhoz V, et al. Evaluation of the efficiency of the Coriolis air sampler for pollen detection in South Europe. *Aerobiologia (Bologna)* 2010;**26**. https://doi.org/10.1007/s10453-009-9152-4

16. Chang CW, Ting YT, Horng YJ. Collection efficiency of liquid-based samplers for fungi in indoor air. *Indoor Air* 2019;**29**. https://doi.org/10.1111/ina.12535

17. Dybwad M, Skogan G, Blatny JM. Comparative testing and evaluation of nine different air samplers: End-to-end sampling efficiencies as specific performance measurements for bioaerosol applications. *Aerosol Science and Technology* 2014;**48**. https://doi.org/10.1080/02786826.2013.871501

18. Bøifot KO, Skogan G, Dybwad M. Sampling efficiency and nucleic acid stability during long-term sampling with different bioaerosol samplers. *Environ Monit Assess* 2024;**196**. https://doi.org/10.1007/s10661-024-12735-7

19. Kesavan J, Sagripanti JL. Evaluation criteria for bioaerosol samplers. *Environmental Sciences: Processes and Impacts* 2015;**17**. https://doi.org/10.1039/c4em00510d

20. Oteros J, Buters J, Laven G, Röseler S, Wachter R, Schmidt-Weber C, et al. Errors in determining the flow rate of Hirst-type pollen traps. *Aerobiologia (Bologna)* 2017;**33**. https://doi.org/10.1007/s10453-016-9467-x

21. Adamov S, Lemonis N, Clot B, Crouzy B, Gehrig R, Graber MJ, et al. On the measurement uncertainty of Hirst-type volumetric pollen and spore samplers. *Aerobiologia (Bologna)* 2024;**40**. https://doi.org/10.1007/s10453-021-09724-5

22. De Sousa NR, Shen L, Silcott D, Call CJ, Rothfuchs AG. Operative and technical modifications to the coriolis® µ air sampler that improve sample recovery and biosafety during microbiological air sampling. *Ann Work Expo Health* 2020;**64**:852–865. https://doi.org/10.1093/ANNWEH/WXAA053

23. Langer V, Hartmann G, Niessner R, Seidel M. Rapid quantification of bioaerosols containing L. pneumophila by Coriolis® µ air sampler and chemiluminescence antibody microarrays. *J Aerosol Sci* 2012;**48**. https://doi.org/10.1016/j.jaerosci.2012.02.001

24. Negron A, Deleon-Rodriguez N, Waters SM, Ziemba LD, Anderson B, Bergin M, et al. Using flow cytometry and light-induced fluorescence to characterize the variability and characteristics of bioaerosols in springtime in Metro Atlanta, Georgia. *Atmos Chem Phys* 2020;**20**:1817–1838. https://doi.org/10.5194/acp-20-1817-2020

25. Zhang PQ, Tan PC, Gao YM, Zhang XJ, Xie Y, Zheng DN, et al. The effect of glycerol as a cryoprotective agent in the cryopreservation of adipose tissue. *Stem Cell Res Ther* 2022;**13**. https://doi.org/10.1186/s13287-022-02817-z
